# Synergistic effects of alkalines, salts, and silica nanoparticles on in-situ surfactant generation from different crude oils in relation to enhanced oil recovery

**DOI:** 10.1038/s41598-026-55523-8

**Published:** 2026-05-31

**Authors:** Ahmadreza Salehi, Abdolhossein Hemmati-Sarapardeh, Mohammad Ranjbar, Mahin Schaffie

**Affiliations:** 1 Department of Petroleum Engineering, Shahid Bahonar University of Kerman, Kerman, Iran; 2 State Key Laboratory of Petroleum Resources and Prospecting, China University of Petroleum (Beijing), Beijing, China; 3 Department of Mining Engineering, Shahid Bahonar University of Kerman, Kerman, Iran

**Keywords:** In-situ, Surfactant, Alkaline flooding, Enhanced oil recovery, Interfacial tension, Wettability, Chemistry, Energy science and technology, Engineering, Environmental sciences, Materials science

## Abstract

The objective of this study was to evaluate the potential of three selected Iranian crude oils for in-situ surfactant generation in relation to alkaline-assisted enhanced oil recovery. The individual and synergistic effects of different alkaline agents, monovalent and divalent salts, and silica nanoparticles (SiO₂) were investigated using interfacial tension and contact angle measurements. In addition, zeta potential (ZP), elemental analysis (EDXA), X-ray Fluorescence (XRF), Hydrogen Nuclear Magnetic Resonance (H-NMR), and Fourier transform infrared Spectroscopy (FTIR) were conducted to assess these effects comprehensively. The results showed that the simultaneous use of two alkalines had a positive effect on interfacial phenomena. The surfactants produced using the heaviest oil in this study (Crude Oil C) have shown the best effects. The interfacial tension was decreased from 35.5 mN/m to 1 mN/m. Furthermore, incorporating silica nanoparticles into the optimal formulation yielded the greatest impact, reducing the interfacial tension to 0.69 mN/m. Additionally, the combination of silica nanoparticles (SiO₂), sodium sulfate, and alkaline agents altered the wettability of mineral samples from oil-wet to water-wet conditions. After 14 days aging times, the contact angle for glass changed from 114 ° to 47 °, and for calcite from 108° to 49°. The results of this study should be considered as an initial screening of oil types for the combined use of alkaline and nanoparticle in enhanced oil recovery operations.

## Introduction

With the expansion of global economic development and the urgent needs of industry, society, and transportation for oil, the demand for this valuable resource is increasing day by day^1,2^. The use of conventional methods has significantly declined, as they are associated with limitations such as high interfacial tension and reduced oil displacement efficiency^3,4^.Therefore, it is essential to employ advanced technologies for enhanced oil recovery (EOR)^5–8^. EOR processes, applied after secondary recovery, include chemical, thermal, microbial methods^9–18^. The implementation of these methods, depending on reservoir conditions, can significantly increase oil recovery, potentially raising the recovery factor to 60–70%^7,19–21^. Chemical EOR include polymer flooding, surfactant flooding, and alkaline flooding^22,23^. They enhance sweep efficiency by reducing the interfacial tension between water and crude oil, increasing the viscosity of the displacing fluid, and improving flow through the reservoir’s microchannels^24,25^. One of the most important chemical EOR methods is alkaline injection, which is considered an attractive option due to its relatively low cost and operational simplicity^26–30^. Unlike surfactant flooding, which is costly and subject to adsorption onto rock surfaces, alkaline flooding does not suffer significantly from these limitations. Overall, alkaline flooding is relatively inexpensive compared to surfactant and polymer flooding and is easier to implement operationally^31–35^.

**Fig. 1:**
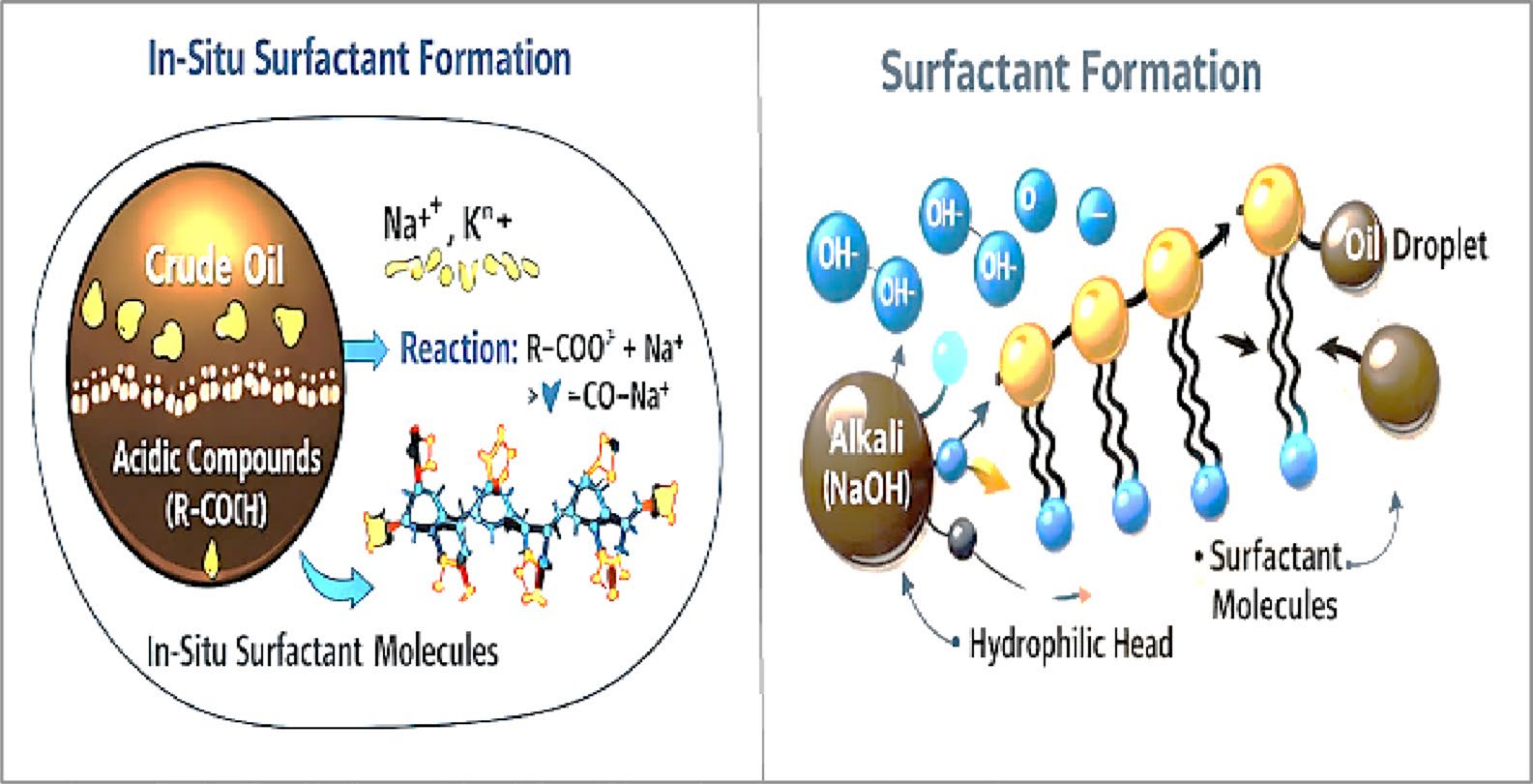
Schematic of in-situ surfactant production by alkaline injection in EOR^45^.

Sodium hydroxide and potassium hydroxide are both strong alkaline agents^17,36^. Sodium hydroxide is generally more favorable from an economic and industrial perspective because it is less expensive, more readily available, more effective at increasing pH, and highly efficient in reducing interfacial tension^37–39^. Potassium hydroxide has similar chemical properties but differs in some physical characteristics^40,41^. Alkaline flooding generates in-situ surfactants through reactions with acidic components present in crude oil. Recovering oil from heavy oil reservoirs with high viscosity is particularly challenging^40,42^. This method is especially effective in oil reservoirs with high acid numbers, where the concentration of organic acids in crude oil is significant^37,43,44^. Figure 1 shows a schematic presentation of surfactant generation by alkaline injection. The reaction between hydroxide ions and the acidic components of crude oil leads to the formation of surfactants^45^.

Alkaline flooding enhances oil recovery through several mechanisms, including interfacial tension reduction, wettability alteration, and emulsification within the porous medium^40,42,46,47^. In reservoirs, oil is trapped in small pores where capillary forces, strongly dependent on interfacial tension, restrict oil movement. As interfacial tension increases, capillary resistance to oil flow increases as well. Therefore, reducing interfacial tension enhances oil mobility and displacement^2,33,43,48^. By generating in-situ surfactants through reactions between crude oil acids and alkaline agents, such as sodium hydroxide, alkaline flooding effectively reduces interfacial tension^2,48,49^. As a result, capillary forces offer less resistance to oil flow, thereby improving oil production and recovery^29,36^. Adhesion forces must also be considered. With reduced interfacial tension, oil droplets can pass more easily through small pore throats, indicating reduced adhesion between oil and rock surfaces^50–53^. Furthermore, alkaline agents, such as sodium hydroxide, through pH control and salinity adjustment, alter reservoir rock wettability from oil-wet to water-wet conditions. In such conditions, oil becomes the continuous phase, favorable mobility ratios are achieved, and oil recovery is significantly enhanced^36,43,54^.

In recent years, research has focused on the combined use of surfactants and nanoparticles with the aim of simultaneously reducing interfacial tension and modifying wettability. Tangparitkul et al^55^. investigated how modified nanoparticles alter the wettability of carbonate reservoirs. They found that positively charged nanoparticles, both alone and in synergy with a surfactant, significantly reduced the contact angle. Laben et al^56^. explored the effect of chemical agents in ASP injection on emulsion stability; their results showed that AOS surfactant increases emulsion stability above 100 ppm but promotes water separation at lower concentrations. Kandiel et al^57^. studied nanoparticle applications in enhanced oil recovery, noting that silica nanoparticles substantially reduced interfacial tension, while hematite nanoparticles maintained good stability across various temperatures. Belhaj et al^11^. focused on surfactant loss due to rock adsorption and oil phase partitioning, concluding that controlling parameters such as temperature, salinity, and rock mineralogy effectively minimized surfactant loss. Adil et al^58^. compared silica nanoparticles modified with Triton X-100 and polyethylene glycol with the Triton X-100-based nanoparticles. Devakumar et al^59^. examined the synergy between silica nanoparticles and anionic surfactants in the presence of monovalent and divalent salts; they found that alkali addition enhanced nanofluid stability and reduced interfacial tension to 0.99 mN/m.

Akpoturi and Ofesi^27^ tested conventional alkalis including sodium hydroxide, potassium hydroxide, and sodium carbonate, as well as a locally produced alkaline derived from palm bunch ash. Their results indicated that oil recovery improved by 66% with sodium hydroxide, 74% with potassium hydroxide, 59% with sodium carbonate, and 64% with the palm bunch ash-based agent. Dutta and Neog^60^ examined the individual and combined effects of alkali, surfactant, and polymer on interfacial tension (IFT) and wettability. The alkali-surfactant binary system lowered IFT from 25.36 to 0.003 mN/m and reduced the contact angle from 112.5° to 55.3°. Alawode and Nekwaya^15^investigated the impact of alkaline nanofluid flooding on interfacial tension (IFT). The combined nanofluid alkali system not only effectively reduced IFT but also increased oil recovery following water injection. Owing to their high surface energy and reactivity, nanoparticles enhance sweep efficiency by migrating through pore throats. Hassan et al^3^. investigated the effect of silica nanoparticles combined with an organic alkali, ethylene diamine, chosen to prevent precipitation and permeability reduction. Increasing alkali concentration reduced IFT from 39.18 mN/m to 8.9 mN/m. Ding et al^61^. studied the influence of three alkalis sodium hydroxide, diethyl amine, and a blend of sodium carbonate with sodium metaborate on IFT and oil recovery through micromodel experiments using heavy Chinese crude oil. Findings revealed that oil displacement is highly dependent on the alkali type and its concentration. IFT values dropped to as low as 0.01 mN/m. Pei et al^62^. studied alkaline flooding for heavy oil recovery using micromodel and sand-pack tests. Proper design of injection parameters was emphasized as critical for effective heavy oil recovery. Esfandiarian et al^63^. investigated the synergistic effects of sodium hydroxide with the anionic surfactant sodium dodecyl benzenesulfonate (SDBS) and the cationic surfactant CTAB on IFT. Increasing sodium hydroxide concentration beyond optimal levels increased IFT in both individual and synergistic systems. The SDBS-sodium hydroxide system showed the best compatibility and IFT reduction. Fakher et al^60^. studied the synergistic effects of three alkalis and two salts. The dissolution of sodium hydroxide in water is an exothermic process that can elevate the temperature of the injected fluid. This reaction forms in-situ surfactants, which are highly effective at reducing interfacial tension. Sodium hydroxide was identified as the most effective alkali for oil recovery efficiency. Saha et al^36^. investigated the different mechanisms during alkaline flooding by using sodium hydroxide. The study concluded that improved oil production results from the combined effects of multiple mechanisms rather than IFT or wettability alone. Honarvar et al^61^. examined the synergistic effects of sodium hydroxide and sodium carbonate with sodium chloride on a light crude oil with an acid number of 0.19 mg KOH/g. Their results showed that this combination reduced the oil-water interfacial tension to below 0.1 mN/m. Rezaei et al^64^. investigated the synergistic effects of the surfactant cocamidopropyl betaine (CAPB) with silica nanoparticles, as well as the synergistic effect of this surfactant with sodium carbonate. The combined effect of the surfactant at an optimal concentration of 250 ppm and nanoparticles at 1000 ppm reduced the interfacial tension from 40 mN/m to 2.5 mN/m. Subsequently, the synergistic effect with sodium carbonate at a concentration of 1000 ppm further decreased the IFT to 0.5 mN/m. Based on these findings, the surfactant-alkali synergy demonstrated superior performance in both oil recovery and interfacial tension reduction compared to the surfactant-nanoparticle synergy.

This study presents the effect of in-situ surfactant generation of light, heavy, and semi-heavy crude oils obtained from Iranian oil fields with different reservoir characteristics. The simultaneous use of these two alkalis rarely investigated together in previous studies offers advantages due to their different physical properties, including lower clay swelling potential, reduced precipitation, less formation damage, and greater stability against scaling compared to their individual use. The individual and combined effects of these alkalis were examined through interfacial tension and wettability experiments on reservoir rock minerals, namely quartz and calcite. After identifying the optimal solution with the most significant interfacial tension reduction, silica nanoparticles were introduced into the system.

## Materials and methods

### Materials

In this study, various chemical materials were utilized. In addition, selected crude oils were employed, and their characteristics are presented in the following sections.

#### Alkalines

To generate in-situ surfactants, two alkaline agent’s sodium hydroxide (NaOH) and potassium hydroxide (KOH) were used.

**Salts**: To investigate the effect of salinity, two types of salts were employed: the monovalent salt sodium chloride (NaCl) and the divalent salt sodium sulfate (Na₂SO₄) both with a purity higher than 99.5%.

#### Nanoparticles

In addition, silica nanoparticles with a purity of 99.5% were used to evaluate the effect of nanoparticles. All chemicals utilized in this study were supplied by Merck, Germany.

#### Crude oils

Three different crude oil samples obtained from Iranian oil fields were used in this study. Oil A is classified as light oil, oil B is considered semi-heavy, and oil C is classified as heavy oil. Table 1 provides colloidal composition API, TAN and density of the crude oils.

**Table 1:** Characteristics of crude oils A, B and C.

Saturation, % wt.	Oil A	Oil B	Oil C
	10.6%	43.5%	36%
Asphaltene, % wt.	5.91%	8%	13.5%
Aromatic, % wt.	54.4%	35.6%	33.2%
Resins, % wt.	24.14%	12.9%	15.1%
API, ° TAN	32.34 0.44	19 2.23	19 2.67
Density (g/cm^3^)	0.84	0.88	0.91

#### Waters

To calculate interfacial tension using **Eq. (2)**, the densities of the primary fluids (distilled water and crude oil) must first be determined. Additionally, the densities of the prepared solutions are required for IFT measurements between oil and other solutions. In addition of distilled water with a density of 0.995 (g/cm^3^), seawater from the Persian Gulf was used for wettability (contact angle) measurements. The composition this water was determined using ICP–MS analysis, and the results are presented in Table 2.

**Table 2:** The main characteristics of Persian Gulf Water.

Ion	Concentration (g/L)	Ionic	Concentration (g/L)
Mg^2+^	1.38	CO_3_^2−^	0
Ca^2+^	0.45	SO_4_^2−^	1
Fe^2+^	< 0.006	HCO_3_^−^	0.12
Si^4+^	0.006	TDS	72
K^+^	0.4	Cl^−^	44
Al^3+^	< 0.001	pH	7.9

#### Rock samples

Calcite (CaCO_3_) and quartz were selected as the rock substrates in this work to assess the alteration of wettability characteristics. These rock samples were obtained from the Geological Department of University. They were selected from the mountains surrounding the city of Kerman in Iran. They were prepared, cut, and polished. X-ray fluorescence (XRF) analysis provided the elemental and compositional data of the rock specimens (Table 3).

## XRF analysis of calcite and quartz rock samples. Note: *loss on ignition

**Table 3:** The main characteristics of Persian Gulf Water.

Calcite		Quartz	
element	Amount (%)	Element	amount (%)
SiO_2_ Al_2_ O_3_ BaO CaO Fe_2_ O_3_ K_2_O MgO MnO Na_2_O P_2_O_5_ LOI^*^	18.29 5.21 0.01 38.91 2.88 1.22 1.09 0.2 0.13 0.07 31.8	SiO_2_ Al_2_ O_3_ BaO CaO Fe_2_ O_3_ K_2_O MgO MnO Na_2_O P_2_O_5_ LOI^*^	95.72 1.63 < 0.01 0.42 0.6 0.37 0.23 0.01 0.07 0.06 0.7

### **Methods**

**Fig. 2:**
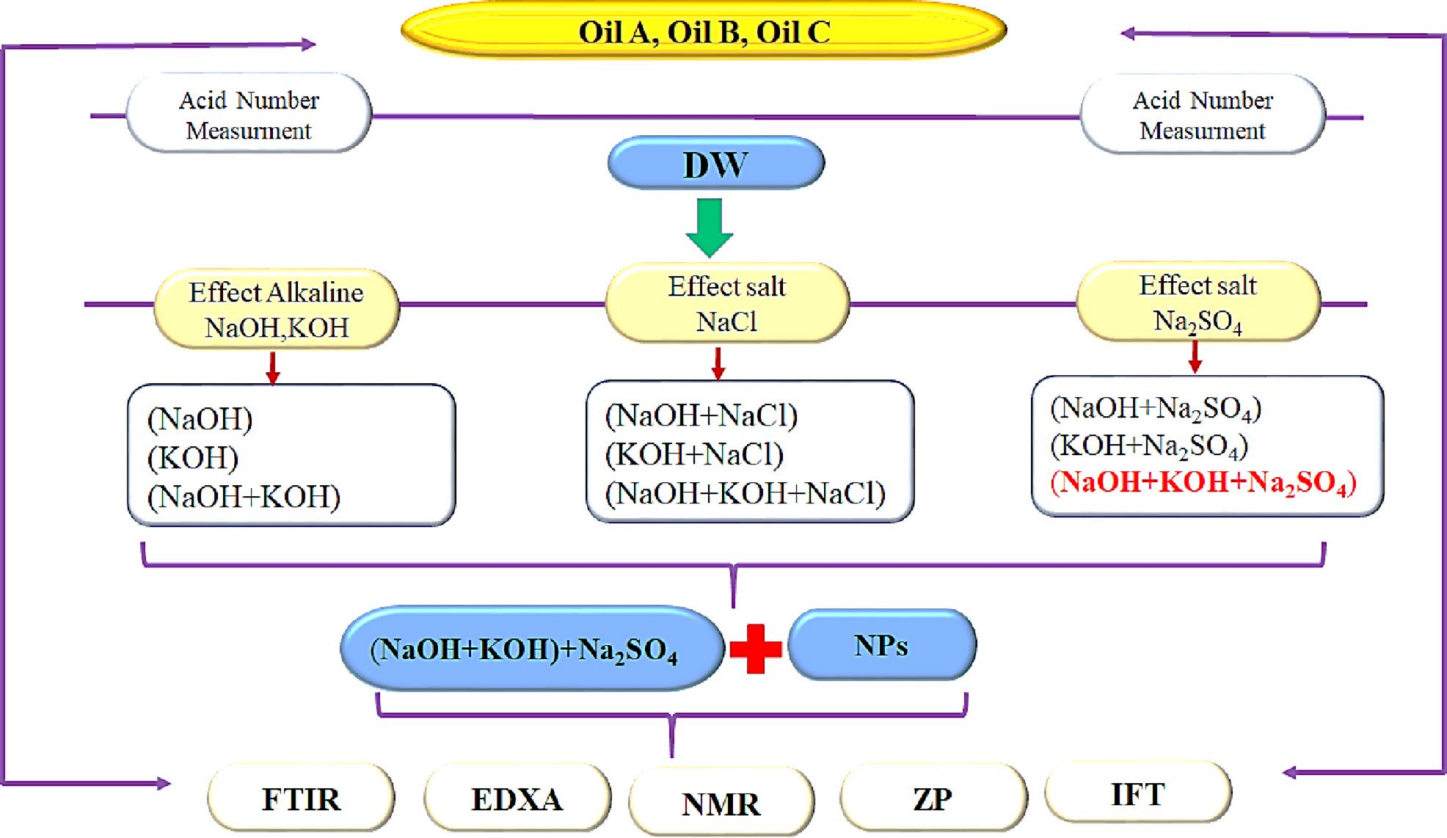
Schematic presentation of interfacial tension tests.

**Fig. 3:**
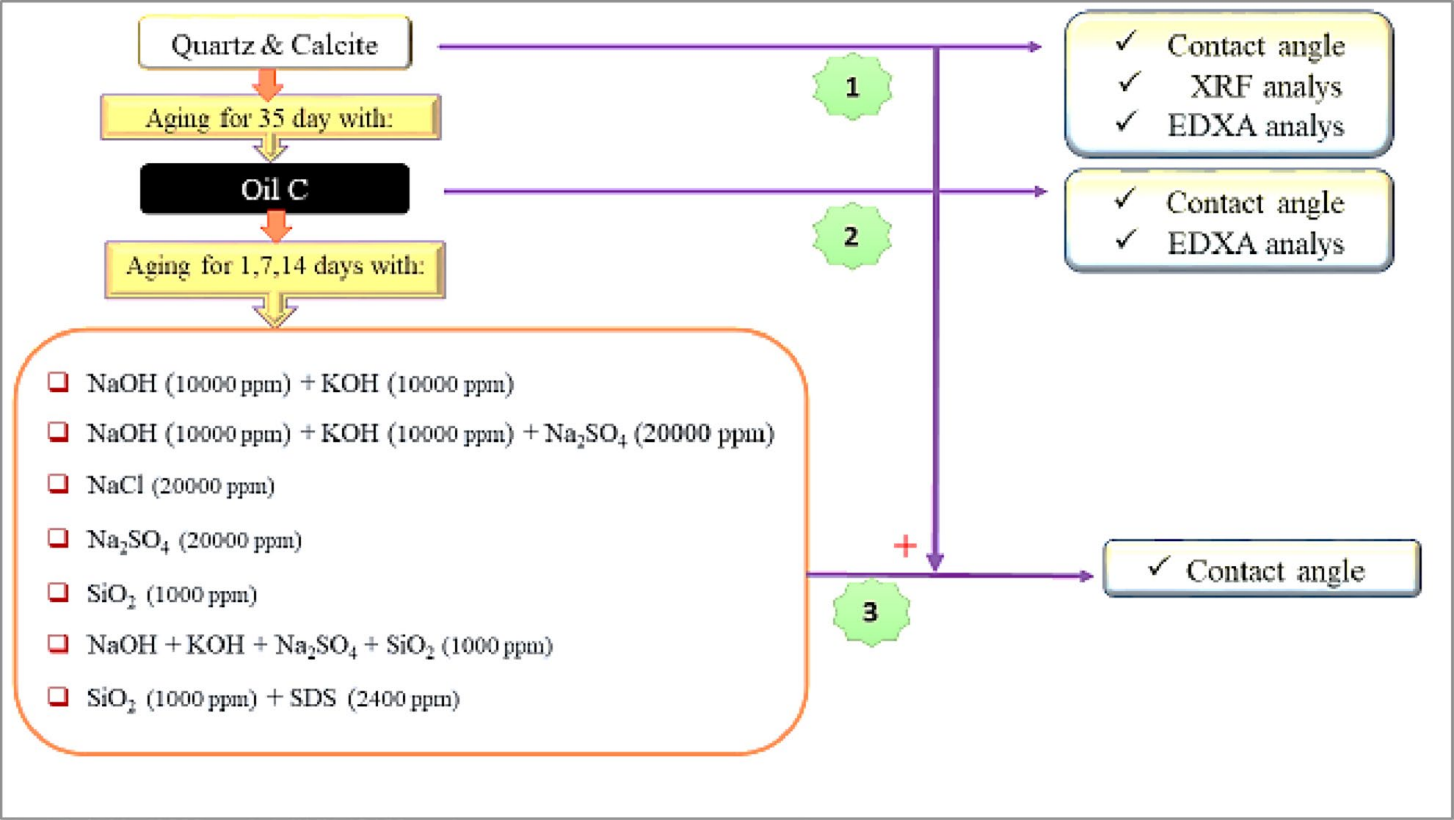
Schematic of presentation of wettability (contact angle) tests.

To practically implement the mechanisms of interfacial tension reduction, wettability alteration, and in-situ surfactant generation, the synergistic effects of alkalis, salts, and silica nanoparticles were evaluated for the selected crude oils. In addition to quantitative results, qualitative analyses, such as zeta potential (ZP), elemental analysis (EDXA), X-ray fluorescence (XRF), hydrogen nuclear magnetic resonance (H-NMR), and Fourier transform infrared spectroscopy (FTIR), were conducted to assess these effects comprehensively. The schematics of IFT and contact angle tests were presented in Figs. 2 and 3. All tests were carried out in triplicate, and the reported values represent the mean of the measurements.

#### measurement of total acid number (TAN)

The total acid number (TAN) is one of the key parameters in petroleum reservoirs. In this research, TAN values were obtained using the ASTM D664 standard protocol. Following the ASTM D664 procedure, a solution was prepared using isopropanol, potassium hydroxide, phenolphthalein, and distilled water. Initially, toluene and isopropanol were mixed at a 1:1 ratio (50 ml toluene and 50 ml isopropanol) and added to a specified amount of crude oil (1–5 g). A 0.1 M potassium hydroxide solution was then added to the oil solvent mixture. A few drops of phenolphthalein were introduced as an indicator until a color change occurred in the alkaline medium, indicating neutralization of the acidic components^65–68^.

To obtain the total acid number, **Eq. 1** was used as follows.

1 $$\:\mathrm{T}\mathrm{A}\mathrm{N}=\frac{0.1\mathrm{*}\mathrm{V}\mathrm{*}56.1}{\mathrm{W}}\:\:$$

where V is the KOH titrant volume (ml) and W is the weight of the crude oil sample (g).

#### Interfacial tension (IFT) measurement

**Fig. 4:**
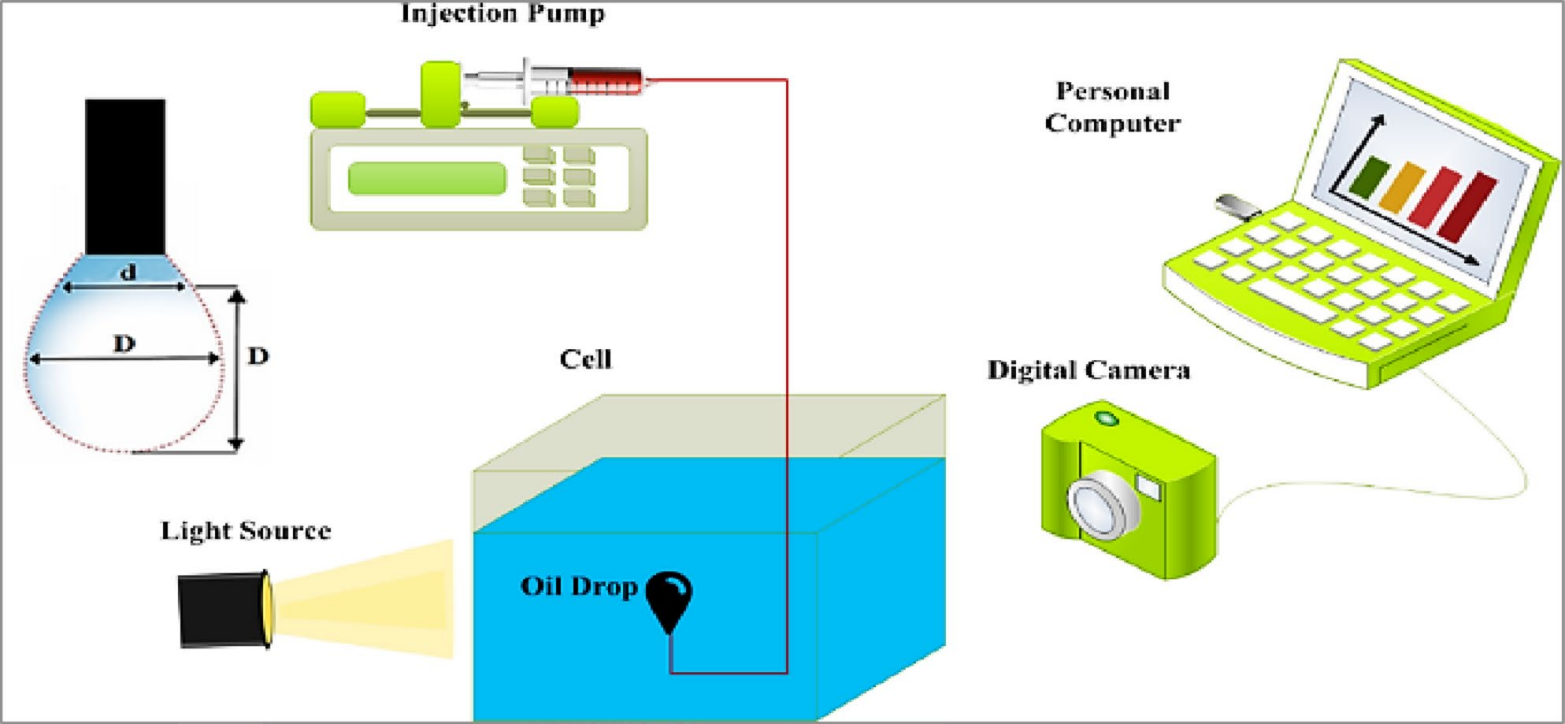
Schematic setup of IFT measurement.

The pendant drop method served to determine the oil-water interfacial tension with a Krüss DSA100 instrument manufactured by Krüss G2, Germany. The schematic setup is shown in Fig. 4^69^. All IFT measurements were conducted under ambient conditions at 25 °C and 1 atmospheric pressure.

For all IFT experiments, 30 ml of distilled water was placed in the measurement cell. In subsequent tests, prepared solutions containing the designated chemicals were used instead of distilled water. The crude oil was continuously injected into the solution at a constant flow rate of 0.2 ml/min via a spinal needle connected to a syringe pump. A digital camera recorded images of the oil drop in its pendant form. The largest (de) and smallest (ds) diameters of the drop were subsequently obtained using Digimizer software. Interfacial tension was obtained from the Young-Laplace relationship as presented in **Eqs. (2)** and **(3)**.

2 $$\:{\upsigma\:}=\frac{\varDelta\:{\uprho\:}\mathrm{g}\mathrm{d}{\mathrm{e}}^{2}}{\mathrm{H}}\:$$

3 $$\:\mathrm{s}=\frac{\mathrm{d}\mathrm{s}}{\mathrm{d}\mathrm{e}}\:$$

σ is interfacial tension (mN/m), Δρ stands for the difference between the density of oil and water. (g/^cm^³), g is gravitational acceleration (9.81 m/s²), de is maximum drop diameter (mm), ds is minimum drop diameter (mm), S = ds/de, 1/H = function of S^70,71^.

#### Contact angle measurement

**Fig. 5:**
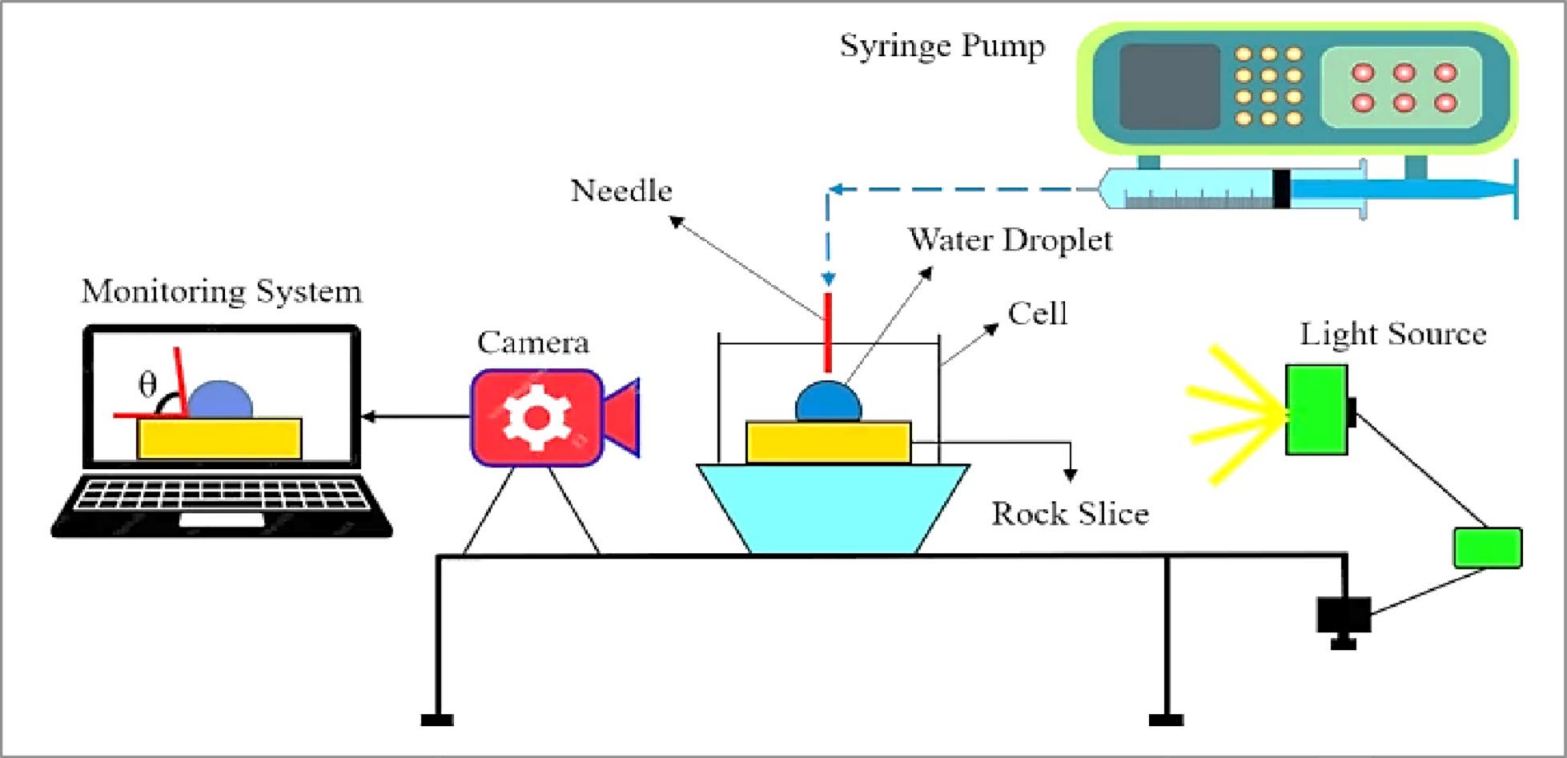
Schematic of wettability setup.

A Krüss DSA100 instrument (Germany) was employed to perform contact angle measurements, as schematically represented in Fig. 5. The mineral sample, previously aged in crude oil for a specified period, was placed beneath the syringe needle. The injection of seawater followed at a constant flow rate of 0.067 ml/min, delivered by a syringe pump. The contact angle experiments were conducted at room temperature (25 °C) and standard atmospheric pressure (1 atm). To investigate contact angle and wettability changes, two minerals calcite and quartz were used along with crude oil 3, which is considered the heaviest oil in this study. All mineral samples were prepared into cubic shapes with dimensions of 3 cm in length, 3 cm in width, and 0.5 cm in height. The aging process began by immersing the minerals in 500 ml of toluene for 5 h in two separate beakers. This step aimed to remove impurities and surface contaminants. Subsequently, each mineral was carefully separated using specialized tweezers and placed in an oven at 50 °C for 7 h. After rinsing to eliminate any residual impurities and ensuring complete surface dryness, the minerals were cleaned with distilled water and then subjected to another 7-hour period in the oven at 50 °C. This procedure effectively removes contaminants, enhancing the accuracy and reducing potential errors in the experiments. Upon completion of this preparation, the quartz and calcite minerals were individually placed in separate reactors, each filled with 25 ml of crude oil. After an immersion period of 35 days, the mineral surfaces became sufficiently oil-wetted and were deemed ready for contact angle measurements. However, several inherent limitations were identified that could influence measurement accuracy. These include edge effects, which cause local variations in the apparent contact angle near sample boundaries. Rock heterogeneity further contributes to this variability, as differences in mineralogical composition across distinct faces of the sample may result in inconsistent wetting behavior. Additionally, surface roughness at the grain scale introduces further constraints; depending on the droplet’s placement, it may settle either on a single grain or across grain boundaries, leading to discrepancies in measured angles. In light of these factors, meticulous attention was given to the steps of cutting, cleaning, and establishing proper contact between the droplet and the solid surface, as these procedures play a critical role in obtaining reproducible and reliable contact angle data.

## Results and discussion

### Spectroscopic characterization of materials

#### FTIR analysis

**Fig. 6:**
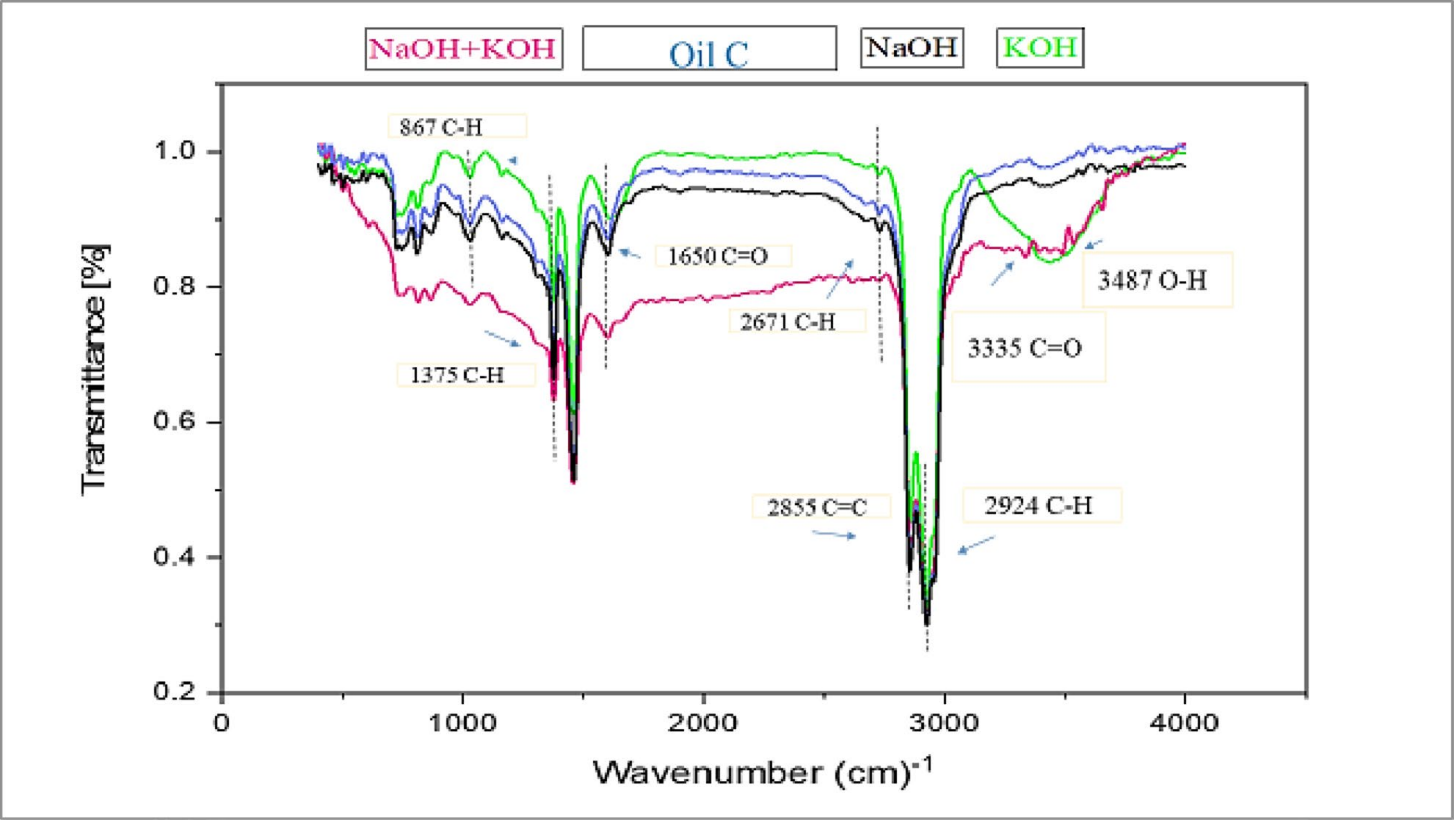
FTIR diagram of the oil and surfactant generated by alkaline.

Crude oil sample 3 was analyzed in its original (blank) state and after in-situ surfactant generation induced by alkaline agents using Fourier transform infrared (FTIR) spectroscopy. In Fig. 6, the red spectrum of the in-situ surfactant features a relatively strong peak at 752 cm⁻¹, which is linked to C–H bonding, indicating that the dominant phase consists of hydrocarbon particles. Furthermore, the peaks appearing at 1459 cm⁻¹ and 2955 cm⁻¹ correspond to C = C bonds^72,73^. In addition, C–H stretching vibrations give rise to the peak observed at 2924 cm⁻¹, representing paraffinic groups in crude oil. The peak at 3335 cm⁻¹ is related to the C = O bond, indicating the presence of strong ketone functional groups, while the peak at 3487 cm⁻¹ corresponds to O–H stretching vibrations^74^. The presence of this bond is expected due to the use of sodium hydroxide and potassium hydroxide, as well as the aqueous nature of the alkaline solution. Moreover, the peaks at 867 cm⁻¹, 1167 cm⁻¹, and 1375 cm⁻¹ are attributed to C–H bonds, indicating stretching vibrations of this group in organic compounds^72–74^. The in-situ surfactant generated by the combination of alkaline solutions exhibits strong absorption peaks in the 2800–3000 cm⁻¹ region, which are mainly attributed to aliphatic-paraffinic C–H stretching vibrations. Accordingly, the alkaline treatment does not noticeably change the fundamental hydrocarbon structure, as C–H groups are relatively stable in the presence of alkali. In addition, a relatively weak peak at around 1650 cm⁻¹ corresponds to the C = O stretching vibration, representing the carbonyl functional group. In the presence of sodium hydroxide and potassium hydroxide in water, these basic compounds react with the organic acids in crude oil, forming water-soluble surfactants. Hence, the reduction in C = O peak intensity relative to the untreated oil verifies that the basic compounds have interacted with the oil’s fatty acids.

#### H-NMR analysis

**Fig. 7:**
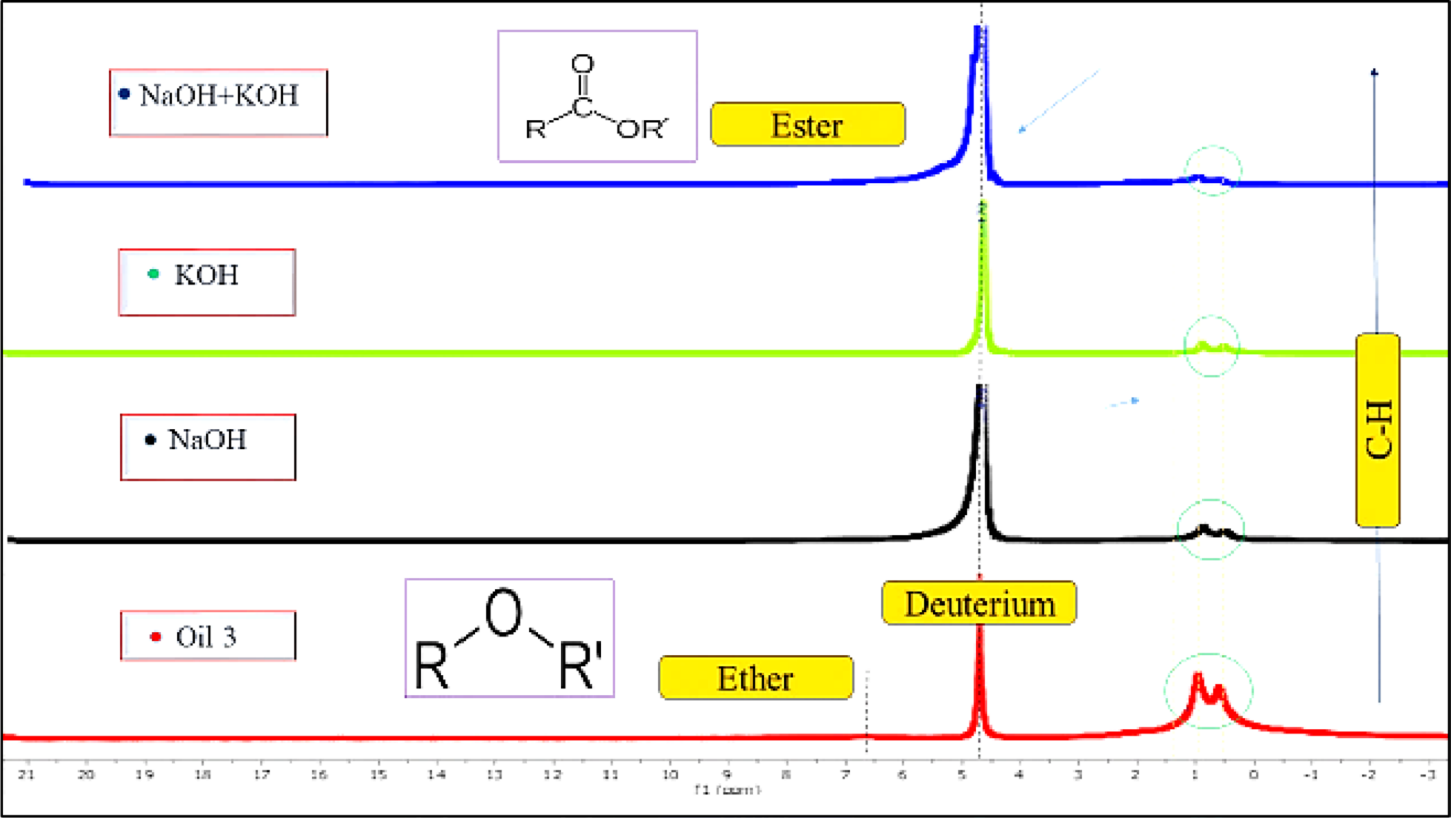
H-NMR analysis of oil and in-situ surfactant a mixture of NaOH and KOH.

To further investigate the chemical components, crude oil sample 3 and the in-situ surfactants generated by different alkaline agents were analyzed using hydrogen nuclear magnetic resonance (H-NMR) spectroscopy. According to the results shown in Fig. 7, the red spectrum displays three main regions of strong peaks. The peaks at 0.96 ppm and 0.58 ppm correspond to hydrogen atoms bonded to carbon, indicating the presence of alkane structures in the crude oil. The peak at 1.1 ppm represents hydrogen atoms associated with carbonyl functional groups. The peak at 4.69 ppm corresponds to the hydrogen of the solvent used in the analysis, deuterium oxide (D₂O). Additionally, a relatively weak peak at 6.61 ppm is observed, which is attributed to hydrogen bonded to carbon adjacent to oxygen, suggesting the presence of light oxygenated derivatives such as ethers^75–77^.

In the blue spectrum, corresponding to the surfactant generated by alkaline agents, the initial peaks at 0.59 ppm and 0.96 ppm similar to the blank sample are related to hydrogen atoms bonded to carbon in alkane (paraffinic) compounds. Furthermore, the peaks at 3.6 ppm and 4.2 ppm indicate the presence of hydrogen atoms in ester functional groups^78,79^. According to these results, adding NaOH and KOH to crude oil, which contains fatty acids and esters, results in the spontaneous formation of surfactants at the site of reaction. Ultimately, neutralization reaction occurs between the alkaline agents and the acidic constituents present in the crude oil. The absence of peaks above 8 ppm indicates the removal of acidic compounds and the lack of characteristic peaks associated with carboxylic acids^80,81^. According to the figure, the red spectrum corresponds to crude oil and is considered the reference. The peaks at approximately 0.58 and 0.96 ppm are assigned to hydrogens bonded to carbon in crude-oil hydrocarbons. The peak at 6.61 ppm is associated with light oxygen-containing compounds such as ethers. In the black spectrum (crude oil treated with sodium hydroxide), similar to the green spectrum (crude oil treated with potassium hydroxide), the initial aliphatic hydrogen peaks decrease in intensity, indicating reduced paraffinic components. Moreover, at around 4.2 ppm, sodium hydroxide chemically interacts with the oil’s inherent naphthenic acids. In the blue spectrum, representing the combined alkali treatment, a notable decrease in hydrogen–carbon compounds are observed. The strong peak at approximately 5.3 ppm suggests that the dual-alkali system causes more extensive decomposition of oil compounds. Additionally, the decreased intensity of the 4.2 ppm peak indicates that the mixed-alkali system reacts more strongly with acidic oil components than either alkali alone.

#### Zeta potential

**Fig. 8:**
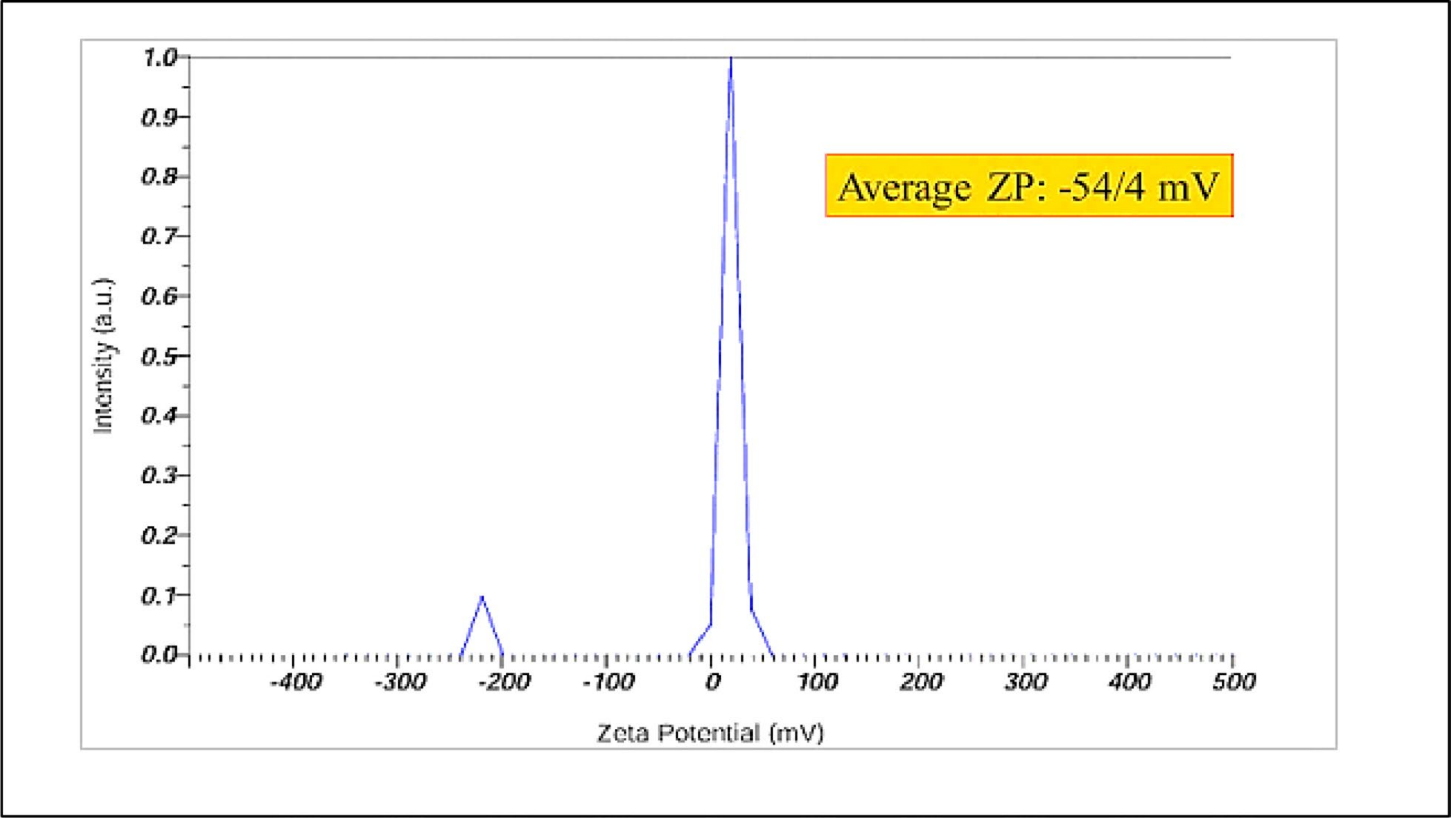
Zeta potential of in-situ surfactant generated using a mixture of NaOH and KOH.

Zeta potential analysis was carried out in order to evaluate the surface charge of the in-situ surfactant generated from the NaOH/KOH combination. As shown in Fig. 8, the measured zeta potential value of − 54.4 mV indicates the formation of an anionic surfactant^82^. Moreover, the magnitude of this electrical charge suggests that the surfactant is stable. Two key elements the elevated asphaltene concentration in the crude oil and a pH above 12 for the alkaline solution are thought to be responsible for stabilizing the anionic surfactant^83,84^.

#### EDXA analysis

**Fig. 9:**
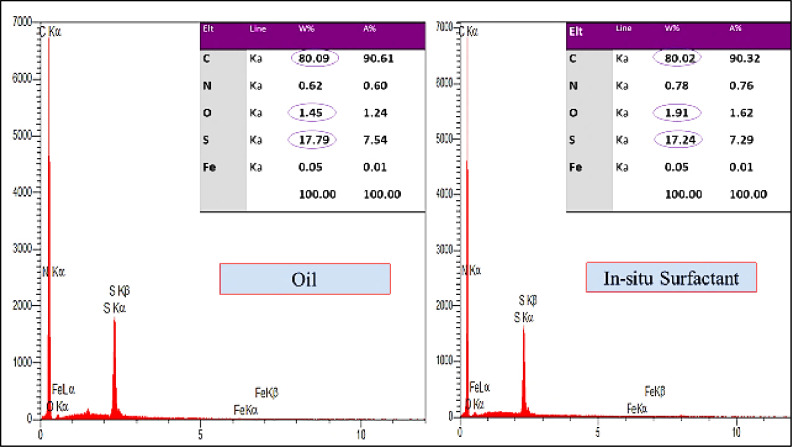
EDXA diagram for oil and surfactant produced by a mixture of NaOH-KOH.

Elemental analysis of crude oil sample 3, both in its original state and with the in-situ surfactant produced by a mixture of NaOH and KOH, was carried out using energy-dispersive X-ray analysis (EDXA), as illustrated in Fig. 9. The elemental composition of crude oil sample 3 shows that carbon, with a content of 80.09%, has the highest intensity, while iron, with 0.05%, exhibits the lowest intensity. In the case of the surfactant produced by alkaline, a noticeable change is observed in the oxygen peak, which increases from 1.45% to 1.91%, indicating the incorporation of oxygen-containing functional groups^85–87^.

**Fig. 10:**
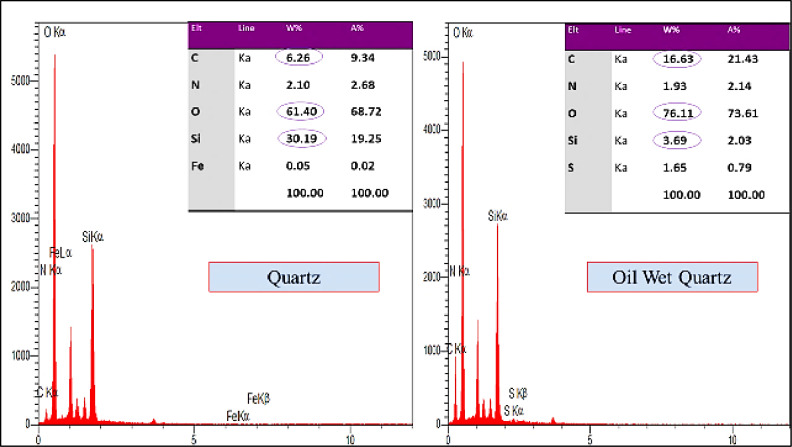
EDXA diagram of quartz before and after aging.

The elemental composition and corresponding spectra of glass (quartz) before oil aging and after oil aging are also presented in Fig. 10. The main elements of quartz, oxygen and silicon (O and Si), exhibit the highest intensities with contents of 61.40% and 30.19%, respectively. The carbon content increases from 6.26% to 16.63%, indicating oil-wet behavior. Additionally, the oxygen and silicon contents change to 76.11% and 3.69%, respectively, reflecting a reduction in the relative proportions of the primary quartz components^88,89^.

**Fig. 11:**
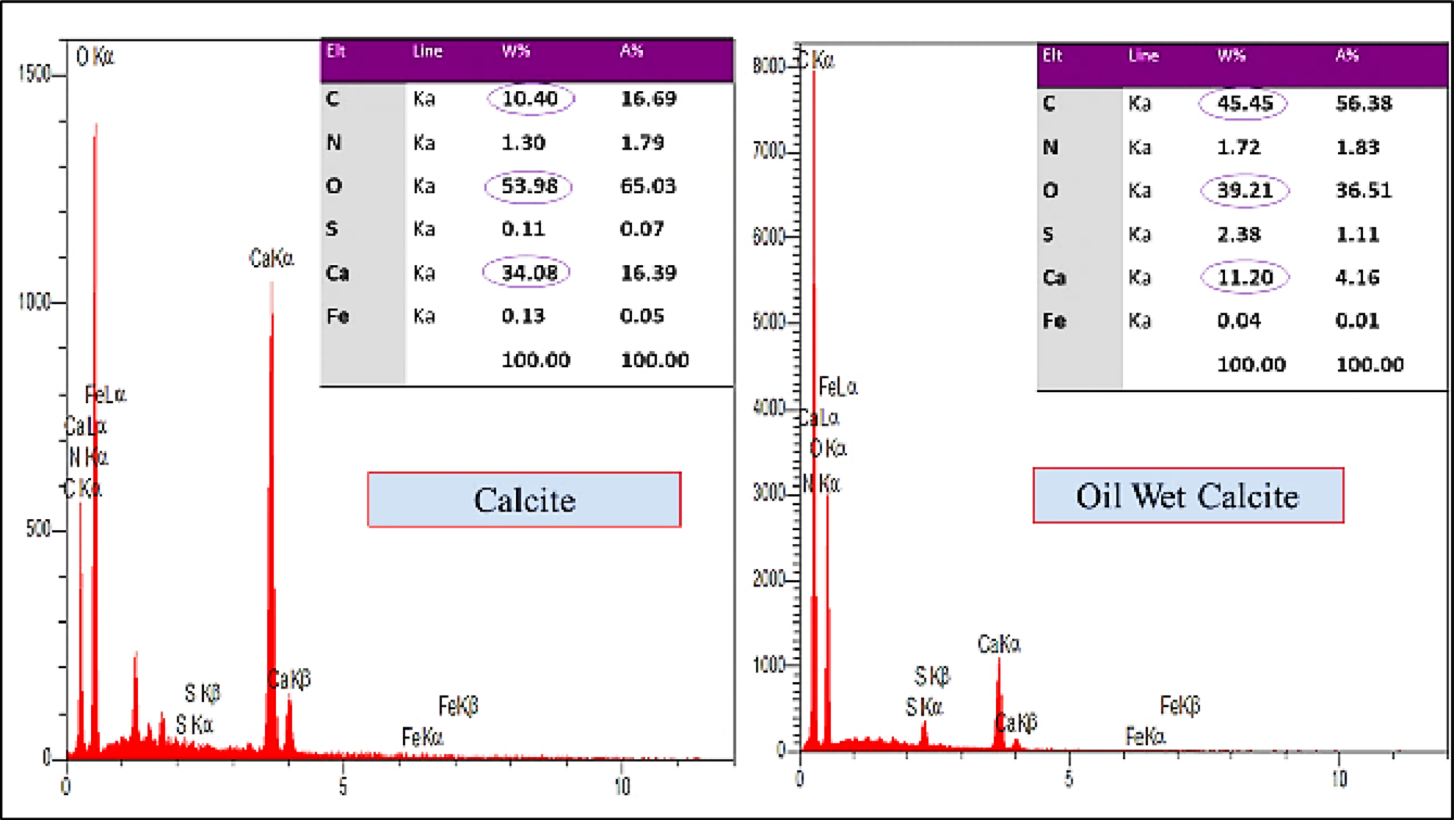
EDXA diagram of calcite before and after aging.

Figure 11 illustrates the elemental composition and spectra of calcite rock before and after contact with crude oil. In the untreated state, before oil exposure, oxygen, calcium, and carbon are the dominant elements with contents of 53.98%, 34.08%, and 10.40%, respectively, indicating that oxygen is the major constituent of the calcite structure. After oil exposure and under oil-wet conditions, the contents of oxygen, calcium, and carbon change to 39.21%, 11.2%, and 45.45%, respectively. The dominant peak corresponding to carbon, along with its significant increase, confirms that the calcite surface has become oil-wet^6,88,90^.

### **Interfacial tension results**

**Fig. 12:**
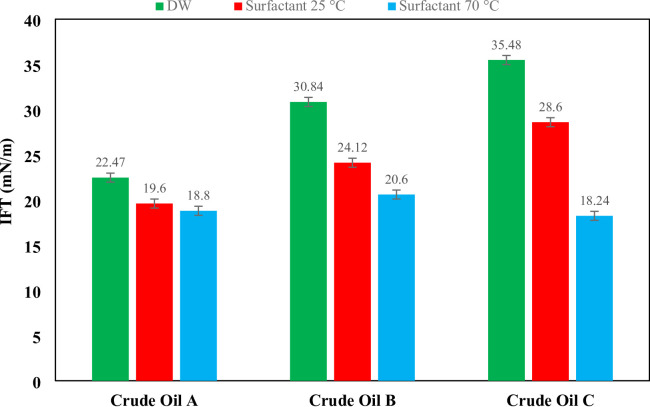
IFT value between DW and crude oil A, B and C, separated after Oil/alkaline contact.

The baseline IFT values between distilled water and crude oil samples A, B, and C are presented in Fig. 12 (calculated using **Eq. 2**). The green bars in the figure represent the IFT between DW and various crude oils. The red bar illustrates the interfacial tension of the oils after the generation of surfactants using NaOH and KOH. Notably, the crude oil subjected to interfacial tension measurement was processed post-surfactant generation. Specifically, upon adding alkali to water, followed by the introduction of crude oil, a surfactant film forms between the water and oil phases. After the surfactant is formed and situated between these two fluids, the oil is separated, and the interfacial tension at 25 °C is measured against distilled water (red bars). The blue bar mirrors the results of the same measurement at the typical reservoir oil temperatures (70 °C) compared to 25 °C. The experimental procedure was identical to the tests at 25 °C, except that both surfactant generation and the subsequent separation of the surfactant-producing oil from the alkaline solution were conducted at a reservoir-equivalent temperature of 70 °C. From the results presented in Fig. 12, it can be pointed out that operation temperature influences both surfactant generation and also IFT-reduction, which are useful effect during alkaline flooding.

#### Effect of alkaline on oil-water interfacial tension

**Fig. 13:**
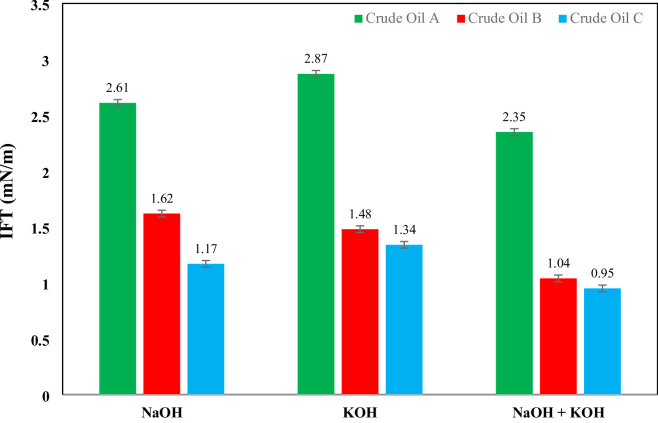
Effect of alkaline (10000 ppm (1 wt%)) on the IFT of oil A, B and C.

As shown in this Fig. 13, sodium hydroxide decreased the IFT of oil A from 22.47 to 2.61 mN/m, that of oil B from 30.84 to 1.62 mN/m, and that of oil C from 35.48 to 1.17 mN/m. Similarly, potassium hydroxide reduced the IFT of oil A to 2.87 mN/m, oil B to 1.48 mN/m, and oil C to 1.34 mN/m. When NaOH and KOH were combined at the same concentration (10,000 ppm), the IFT values further reduced to 2.35 mN/m for oil A, 1.04 mN/m for oil B, and 0.95 mN/m for oil C. These findings point to the conclusion that the alkaline mixture had a more substantial effect, particularly for the heavy oil (oil C), compared to the individual alkaline solutions. Figure 13 presents results obtained through a different experimental approach, adhering to standard procedures for pendant drop IFT measurements. In this setup, oil droplets were added to distilled water (base test) and to distinct and mixed alkaline solutions. A fundamental difference from Fig. 12 is that the oils in Fig. 13 were not subjected to the pre-treatment of alkali contact followed by separation. Instead, Fig. 13 reflects standard, direct room-temperature IFT tests conducted with various crude oils and alkaline solutions. The enhanced performance of the combined alkaline solution suggests improved in-situ surfactant generation, which can significantly contribute to enhanced oil recovery (EOR).

#### Effect of salts

Previous studies have looked at how salinity impacts crude oil-brine IFT. Reported results vary due to differences in crude oil composition, particularly acidic components, asphaltene content, and natural surfactants. In addition to salinity, temperature, pressure, and total acid number (TAN) significantly affect IFT behavior^91–93^. Experimental findings indicate that salts reduce IFT up to an optimal concentration. At this point, ionic species at the oil-water boundary reach equilibrium, leading to maximum interfacial activity. Beyond this optimal salinity, further increases result in a gradual increase in IFT. According to the Gibbs adsorption isotherm, increasing salinity enhances density differences between oil and water phases, contributing to higher IFT values^11,94,95^.

**Fig. 14:**
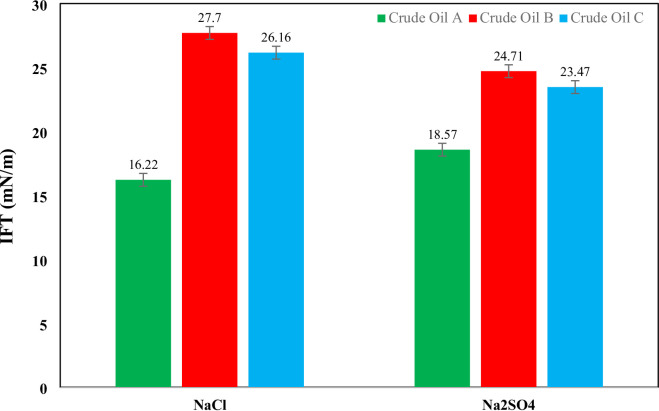
Effect of various salts on IFT: a comparative plot for oil samples A, B, and C.

In the present study, monovalent sodium chloride and divalent sodium sulfate were evaluated at an optimum concentration of 20,000 ppm, both individually and in combination with alkaline agents. Low salinity conditions promote the aggregation of asphaltene species at the boundary between oil and brine, creating unstable and less flexible interfacial films due to steric hindrance. At high salinity, ionized crude oil molecules aggregate and precipitate due to electrostatic repulsion. Moderate salinity can enhance solubility and interfacial activity; however, excessive salinity reduces solubility after reaching a maximum value^49,91,95,96^. As shown in Fig. 14, compared the results presented in Fig. 12, the addition of sodium chloride to DW reduced the IFT to 16.22 mN/m for oil A, to 27.7 mN/m for oil B, and to 26.16 mN/m for oil C. Additionally, sodium sulfate further reduced the IFT to 18.57 mN/m for oil A, 24.7 mN/m for oil B, and 23.47 mN/m for oil C. Compared to NaCl, Na₂SO₄ contains divalent sulfate anions with a larger ionic size and stronger ionic interactions, facilitating more effective interaction with crude oil components and resulting in greater IFT reduction^97,98^.

#### Synergy between salt and alkali agents

**Fig. 15:**
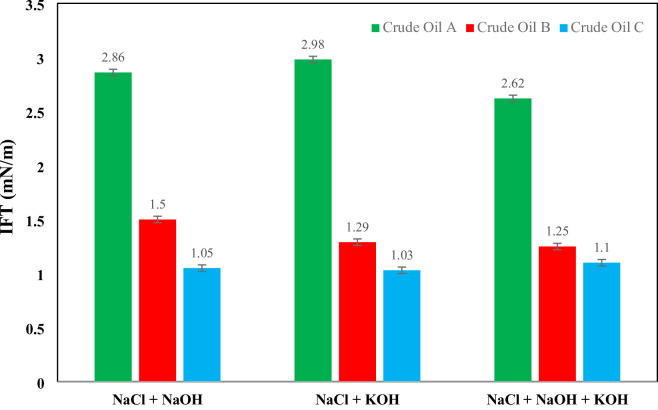
Comparison of synergistic alkaline and NaCl effects on IFT for all crude oils.

To evaluate the synergy between salt and alkali agents, solutions were prepared using 20,000 ppm salt and 10,000 ppm alkali. The combination of sodium chloride with NaOH and KOH produced lower IFT values than individual alkali-salt systems, as shown in Fig. 15. The IFT decreased to 2.62 mN/m for oil A, 1.25 mN/m for oil B, and 1.10 mN/m for oil C.

**Fig. 16:**
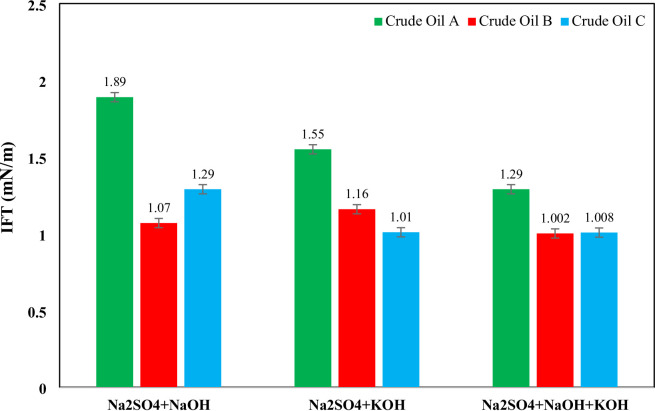
Comparison of synergistic alkaline and Na_2_SO_4_ effects on IFT for all crude oils.

As shown in Fig. 16 for sodium sulfate combined with alkaline agents, the IFT results were further reduced to 1.29 mN/m for oil A, 1.002 mN/m for oil B, and 1.008 mN/m for oil C. These results demonstrate that the combined salt-alkali systems outperform individual components, with the divalent sodium sulfate showing a stronger synergistic effect than sodium chloride. The effect was particularly significant for heavy oil C^99–101^.

The improved effectiveness of sodium sulfate stems from its greater ionic charge, which compresses the electrical double layer at the oil-water interface, decreases electrostatic excretion between acidic molecules, and promotes their accumulation at the interface^102^. This mechanism enhances interfacial film compaction, resulting in lower IFT values. Based on comparisons with baseline IFT measurements, the sodium sulfate-alkali combination can be considered the optimal formulation for IFT reduction^103,104^. Compared to sodium chloride, the synergy of alkaline conditions and sodium sulfate provides significantly superior inhibition in corrosion, stabilization, and coagulation processes by forming less soluble mineral phases and the developing protective surface layers.

#### Effect of nanoparticles

**Fig. 17:**
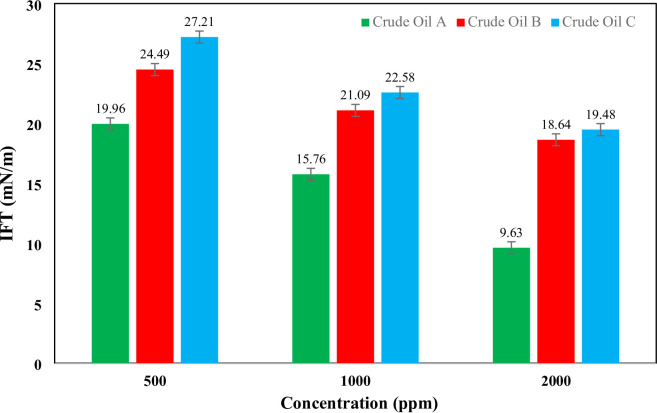
Comparison of NPs effects on IFT for all crude oils.

In this study, silica nanoparticles (SiO₂) were used at concentrations of 500 ppm, 1000 ppm, and 2000 ppm to evaluate their effect on interfacial tension (IFT). Due to their unique structural properties and high adsorption and dispersion capability, increasing nanoparticle concentration is expected to reduce IFT further. However, excessive nanoparticle usage is not recommended due to environmental and economic considerations. The interfacial tension behavior of water and crude oil in the presence of nanoparticles is depicted in Fig. 17.

Nanoparticles contribute to enhanced oil recovery through several mechanisms, including disjoining pressure generation, IFT reduction, wettability alteration, asphaltene stabilization, crude oil viscosity reduction, and injected fluid viscosity enhancement^58,105–108^.

One of the primary mechanisms involves nanoparticle adsorption onto reservoir rock surfaces, which modifies surface properties and alters oil-water contact angles, thereby improving oil detachment from the rock surface^105,106,108,109^. Nanofluids form wedge-shaped structural layers on rock surfaces, generating structural disjoining pressure that separates oil droplets and enhances recovery. Nanoparticles also contribute to a better mobility ratio by thickening the displacing fluid and potentially thinning the crude oil, which leads to improved sweep efficiency and enhanced EOR outcomes^61,105,106,109^.

#### Synergistic effect of nanoparticles with salt and alkali

**Fig. 18:**
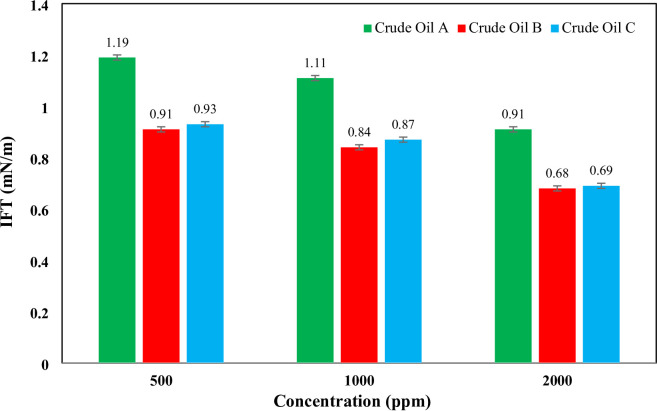
Comparison of nanoparticle and (NaOH/KOH/Na₂SO₄) effects on IFT for all crude oils.

Under the previously identified optimal condition (sodium sulfate combined with mixed alkalis), silica nanoparticles were added at concentrations of 500, 1000, and 2000 ppm. As illustrated in Fig. 18, increasing nanoparticle concentration led to a further reduction in IFT. At 2000 ppm, nanoparticles reduced the IFT to 0.91 mN/m for oil A, 0.68 mN/m for oil B, and 0.69 mN/m for oil C. Compared with baseline values, nanoparticles exhibited the most significant IFT reduction for heavy oil C. The reason for this behavior lies in nanoparticle buildup at the oil-water interface, which both lowers the interfacial free energy and reinforces interfacial stability^106,107,109^.

The mechanisms responsible for improved oil recovery during alkaline-surfactant-nanoparticle flooding are investigated in this study. In-situ surfactant generation via saponification of oil organic acids with alkali (NaOH, KOH) is enhanced by elevated pH, which increases electrostatic repulsion and promotes surfactant packing at the oil-water boundary. Sulfate ions aid in stabilizing the surfactant arrangement. Negatively charged nanoparticles, adsorbing to the interface under alkaline conditions, form a nanostructured layer that orients the generated surfactants and acts as Pickering emulsion agents, further reducing interfacial tension (IFT). Nanoparticles also lower surface free energy and promote oil film detachment from rock surfaces by enhancing electrostatic repulsion and weakening rock-oil adhesion, thereby reducing the contact angle and improving water flow.

**Fig. 19:**
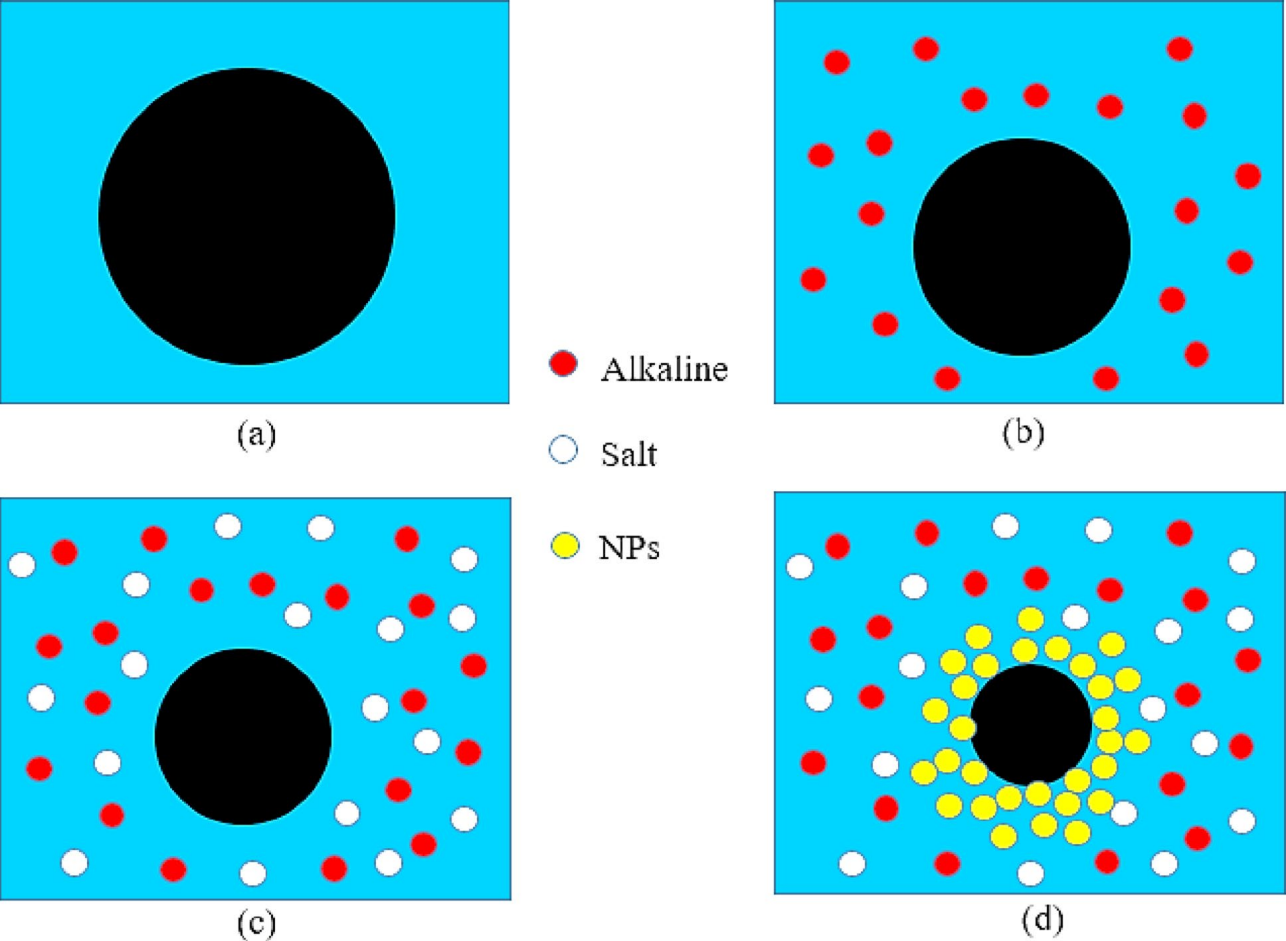
Schematic mechanism of IFT reduction with synergistic of alkaline, salt and NPs.

The process of in-situ surfactant generation is initiated by adding alkali to the organic acids present in crude oil. This alkalization increases pH, leading to greater electrostatic repulsion. Sulfate ions play a crucial role by managing the electrical double layer’s thickness, which enhances the interaction between alkali and organic acids at the oil-water boundary. This interaction promotes the formation of a more densely packed surfactant layer. Nanoparticles, under alkaline conditions, adsorb onto the interface with a significant negative charge, establishing a nanostructured layer and facilitating improved orientation of the synthesized surfactants^11,49^. The combined action of these components results in a stable and dense interfacial film, with the well-oriented surfactants and nanoparticles stabilizing the interface^110^. Specifically, crude oils with high acid numbers possess carboxylic acids that react with alkaline solutions to form soaps. These amphiphilic surfactant molecules then migrate to and stabilize the oil-water boundary, drastically lowering interfacial energy and, consequently, interfacial tension^110,111^. Asphaltene, being the heaviest and most polar oil constituent, also preferentially locate at the oil-water boundary. Their polar functional groups lend them stabilizing attributes. In the presence of in-situ surfactants, asphaltene contribute to strengthening the interfacial surfactant layer, potentially via hydrogen bonding, leading to increased density and further reduction in interfacial tension. Figure 19 illustrates the schematic mechanism of IFT reduction through the synergistic effect of alkali, salt, and nanoparticles. In each step, the surface tension is progressively reduced with the addition of each new component.

**Fig. 20:**
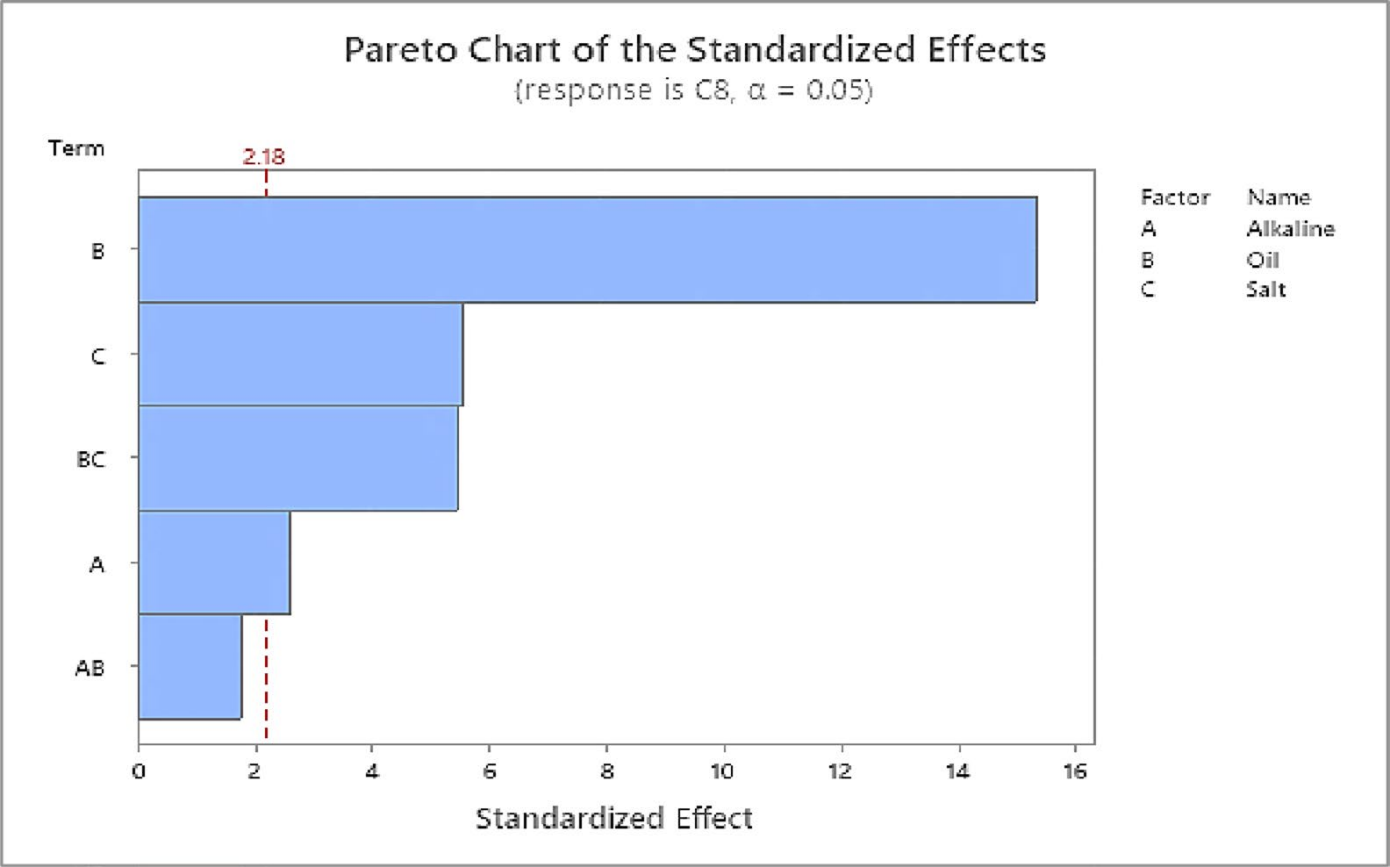
Pareto chart of standardized effects for IFT tests.

The Pareto chart in Fig. 20 was used to evaluate the parameters influencing IFT experiments. According to this analysis, oil and salt exhibited the greatest impact on the experimental outcomes. Although their effects were comparatively smaller, alkaline concentration was also identified as a statistically significant factor contributing to the results. In this study, the key parameters influencing the outcome were alkali type, salt concentration, and crude oil type. Beyond their individual effects, the interactive effects among these parameters are also depicted. Based on the interfacial tension (IFT) results, crude oil type emerged as the factor with the most substantial individual impact on the quantitative results. This indicates that the inherent properties and characteristics of the crude oil were more influential than the specific salt or alkali employed. Following crude oil type, salt type demonstrated a more favorable effect based on the obtained results. Another significant parameter is the combined effect of crude oil and salt, highlighting that both oil and salt exert considerable influence on interfacial tension, both independently and synergistically. The utilization of this Pareto chart is predicated on the computed results and the chosen model for the data. Consequently, crude oil type, salt type, and their interaction are identified as crucial parameters impacting interfacial tension, according to their observed effects.

Furthermore, the results of the analysis of variance (ANOVA) are provided in the corresponding Table 3, where each parameter has been comprehensively evaluated and discussed in detail.

**Table 4:** Analysis of variance for IFT test.

source	DF	Adj SS	Adj MS	F-Value	*P*-Value
Model	14	12.1557	0.86827	30.28	0.000
Linear	6	10.2018	1.70030	59.59	0.000
Alkaline	2	0.2965	0.14823	5.17	0.024
Oil	2	8.7044	4.35222	151.77	0.000
Salt	2	1.2009	0.60043	20.94	0.000
2-Way Interaction	8	1.9539	0.24424	8.52	0.001
Alkaline*Oil	4	0.2812	0.07030	2.45	0.103
Oil*Salt	4	1.6727	0.41818	14.58	0.000
Error	12	0.3441	0.02868		
Total	26	12.4998			

**Fig. 21:**
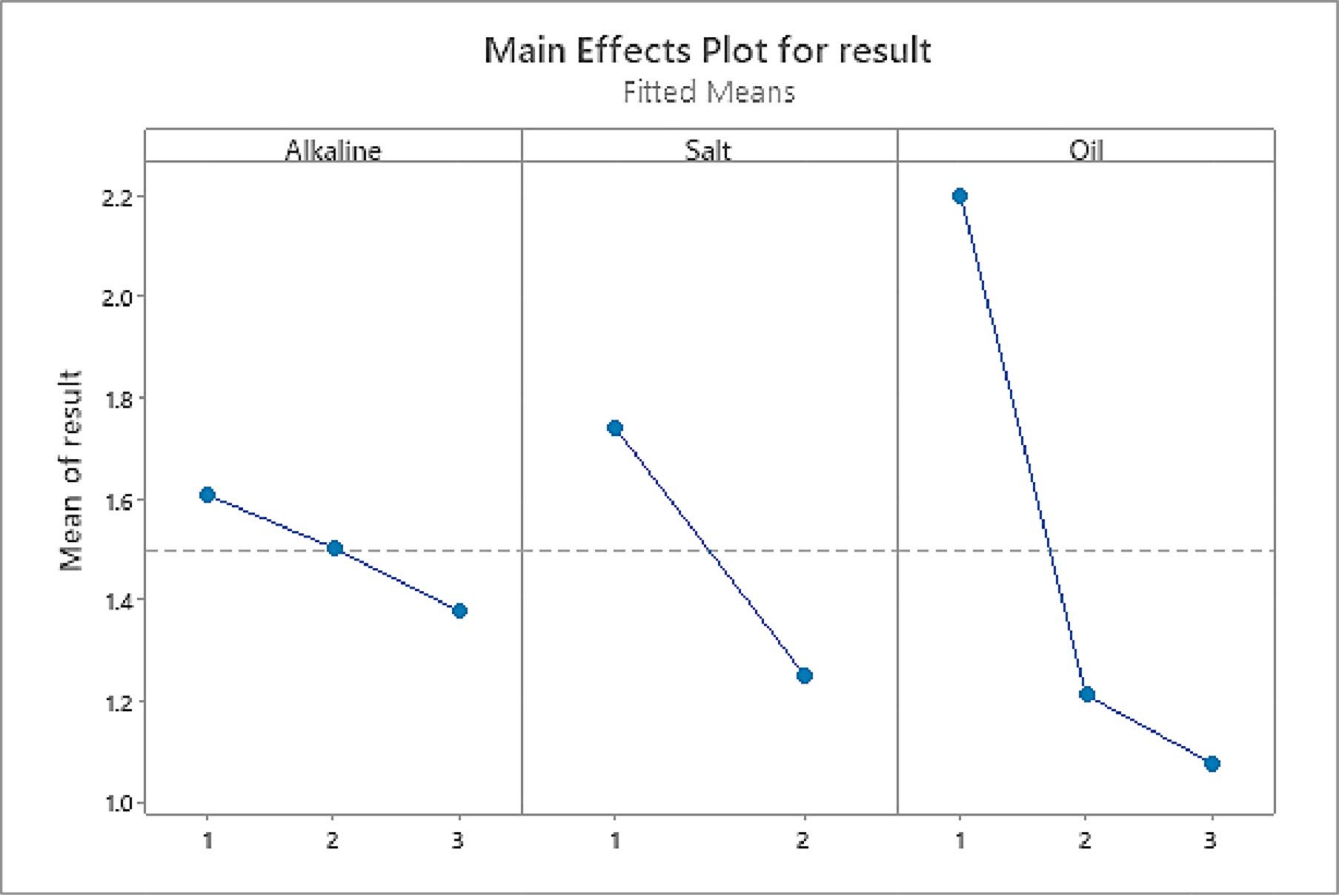
Graph illustrating the effective parameters influencing the interfacial tension experiments.

In Fig. 21, based on the experimental design carried out in this research, the effective parameters influencing interfacial tension are presented. According to the results, the third level of alkaline corresponding to the mixture of sodium hydroxide and potassium hydroxide demonstrated the most significant effect among the alkaline levels. For the salt factor, the second level, namely Sodium sulfate, showed the best performance. Finally, among the oil types, the third-level oil C exhibited the most favorable impact on interfacial tension. The main effects of the investigated factors across their respective levels are also shown. The experimental design involved three levels for the alkali parameter, two levels for salt, and three levels for crude oil type. Observing the trends in Fig. 21, the most favorable outcome for the alkali parameter was achieved at the third level, while the second level proved optimal for salt. For crude oil type, the third level yielded the best results. Specifically, the third alkali level represents a combination of specific alkalis, the second salt level corresponds to the divalent salt sodium sulfate, and the third crude oil level signifies the heaviest crude oil investigated in this study. These specific settings are identified as the most effective and influential factors for achieving the desired interfacial tension reduction.

### Wettability (contact angle measurement)

The wettability behavior of surfaces exposed to various phases can be quantitatively assessed using contact angle measurements. Reservoir rocks are naturally water-wet; however, deposition of heavy hydrocarbons on the rock surface may alter wettability toward oil-wet conditions. After two to four weeks of aging, the wetness condition becomes stable^112^.

**Fig. 22:**
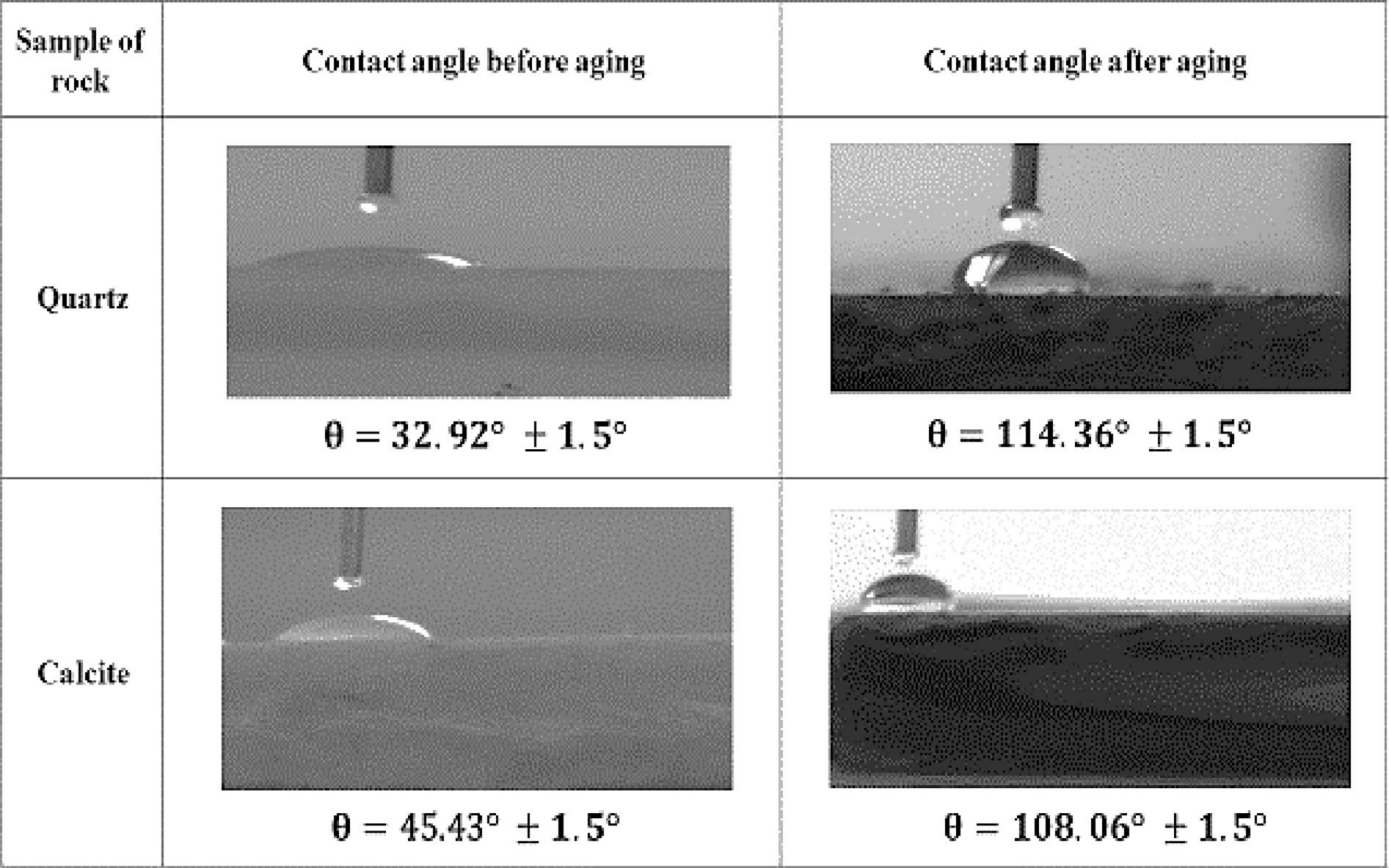
Contact angle for quartz and calcite before and after aging in oil with seawater.

In this study, quartz and calcite minerals were used along with alkaline agents, salts, silica nanoparticles, and heavy oil C (the heaviest oil sample). The minerals were aged with oil for 35 days, and contact angle measurements were conducted using Persian Gulf seawater at three different time intervals. In Fig. 22, the contact angle for both minerals, quartz and calcite, was measured under three conditions: without exposure to crude oil and after exposure to crude oil using seawater.

#### Effect of Alkali

**Fig. 23:**
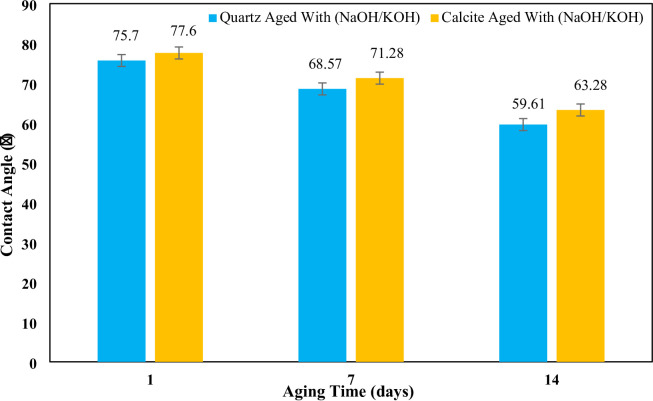
Contact angle response of quartz and calcite to alkaline treatment.

After 14 days, as shown in Fig. 23 the combined NaOH and KOH solution decreased the contact angle from 114.36° to 59.61° for quartz and from 108.09° to 63.28° for calcite, indicating a shift of oil-wet to water-wet conditions.

In alkaline environments, mineral surface groups (Si-OH in quartz and CO₃²⁻ in calcite) undergo deprotonation, increasing the negative surface charge. This enhances electrostatic excretion between the rock surface and polar oil components, promoting water wettability. Hydroxide ions (OH⁻) may also competitively adsorb on the rock surface, displacing previously adsorbed oil components and weakening the oil-wet layer^59,93^.

#### Effect of Salts

**Fig. 24:**
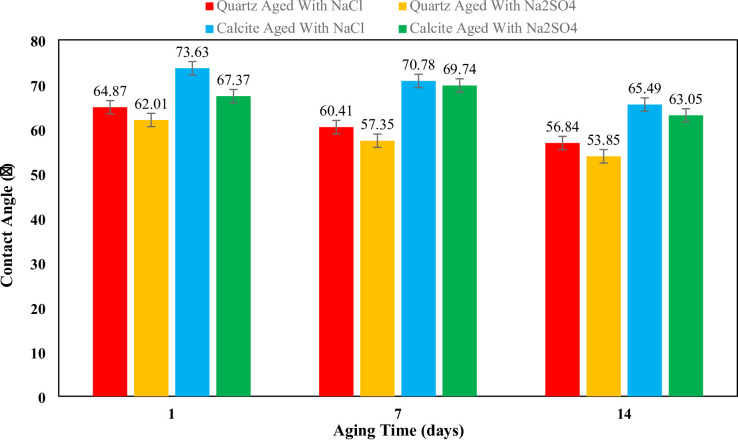
Comparative diagram of salt effects on contact angle: quartz versus calcite.

As shown in Fig. 24, for sodium chloride, the contact angle after 14 days decreased to 56.84° for quartz and 65.49° for calcite. For sodium sulfate, the angles further decreased to 53.85° and 63.05°, respectively.

**Fig. 25:**
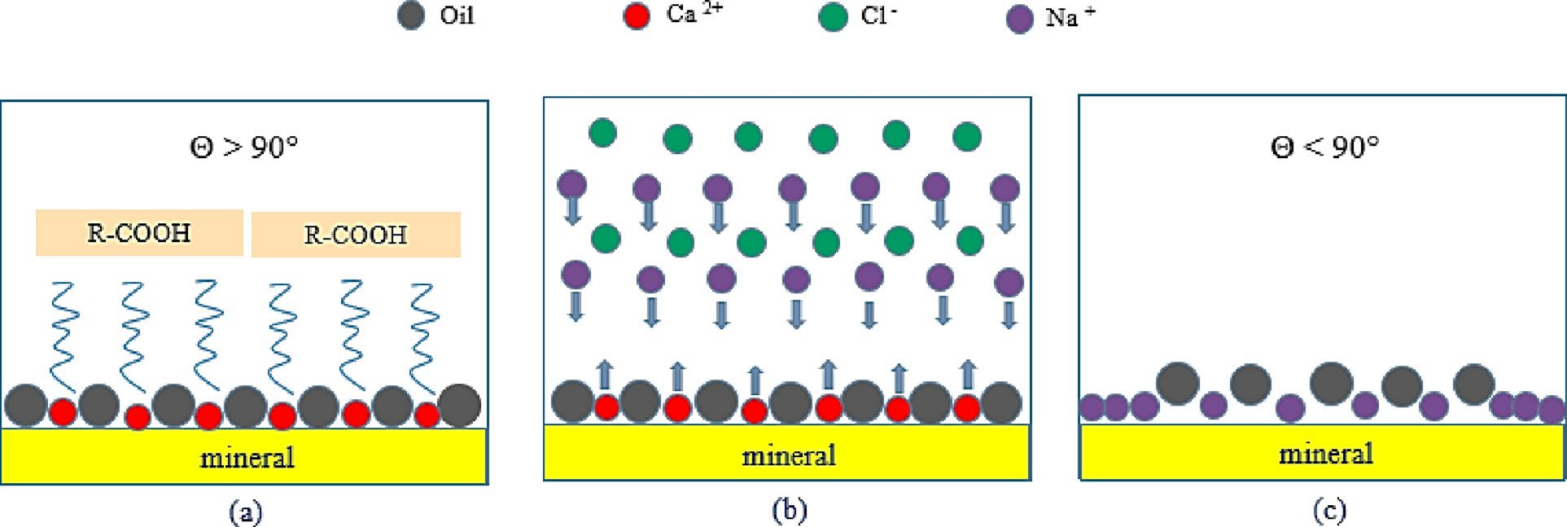
Schematic of salt effects on contact angle: quartz versus calcite: (a) the initial condition where oil adheres to the surface via ionic bridging; (b) the ion exchange process between sodium ions from the aqueous phase and calcium ions on the mineral surface; and (c) final state, characterized by oil detachment and a water-wet surface.

The influence of salt on the wettability of minerals is illustrated schematically in Fig. 25. In low-salinity water injection, the primary mechanism is the substitution of bridging divalent ions with monovalent ions, leading to physical detachment of oil from the rock surface. Monovalent NaCl weakens weak bonds by reducing ionic strength but cannot effectively break Calcium bridges. In contrast, divalent sodium sulfate, due to sulfate ions adsorbing onto carbonate surfaces and sodium reacting with oil acids, significantly reduces the contact angle^113,114^. Ions in solution interact with surface functional groups (Si-OH in quartz and carbonate groups in calcite), altering the adsorption behavior of polar oil components. Variations in salinity can remove organic acids from the rock surface, which in turn alters wettability to become more water-wet^100,115^.

#### Effect of Nanoparticles

**Fig. 26:**
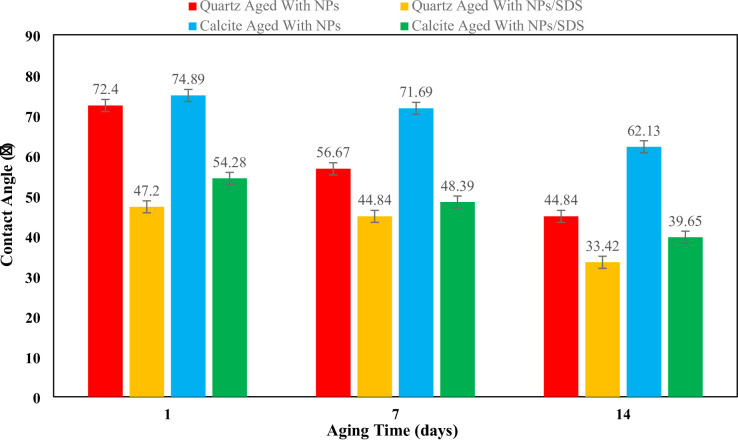
Wettability alteration of the NPs and SDS on the contact angle for quartz and calcite.

The use of silica nanoparticles significantly influenced wettability alteration. As shown in Fig. 26, at a concentration of 1000 ppm, after 14 days, the contact angle reduced to 44.84° for quartz and 62.13° for calcite. Furthermore, due to the combined effect of 1000 ppm silica NPs and 2400 ppm sodium dodecyl sulfate (SDS) surfactant, the contact angle decreased after 14 days to 33.42° for quartz and 39.65° for calcite. Adsorption of nanoparticles on quartz and calcite surfaces, forms a hydrophilic nanoscale layer that prevents direct oil–rock contact and increases water affinity. Furthermore, nanoparticles modify surface free energy and, in some cases, increase nanoscale roughness, contributing to contact angle reduction^105–109^.

#### Synergy between salt and alkali agents

**Fig. 27:**
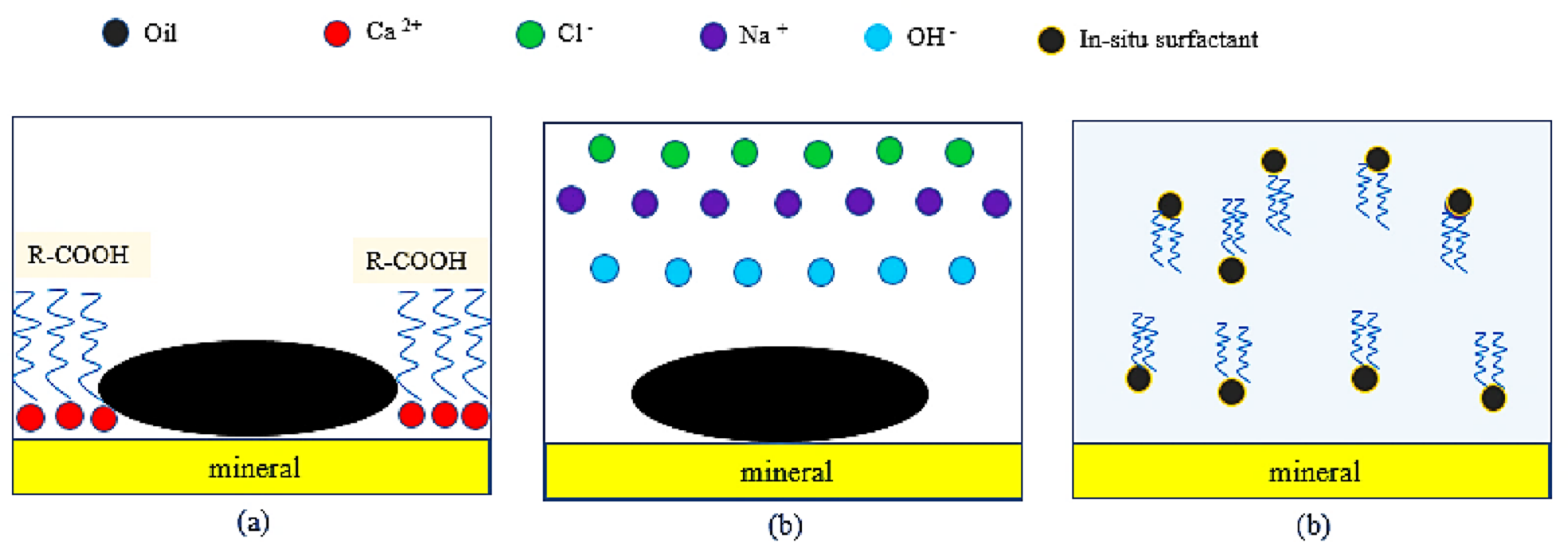
Schematic representation of the synergy effect of alkali and salt on mineral wettability: (a) initial strong oil wet state (b) combined of alkaline-salt injection (c) final strong oil wet state.

Consistent with IFT reduction results, the combined alkali-salt system also positively affected wettability. In sandstones, the main mechanism of alkali and salt synergy is multi-component ion exchange and increased surface negative charge, resulting in mineral water-wetness. Alkali converts naphthenic acids into surfactants, while salt further completes oil detachment by raising the rock surface negative charge and compressing the electrical double layer, ultimately yielding a water-wet surface and a stable oil-in-water emulsion^59,101,114^. The schematic effect of combined alkali and salt on mineral wettability is observable in Fig. 27.

**Fig. 28:**
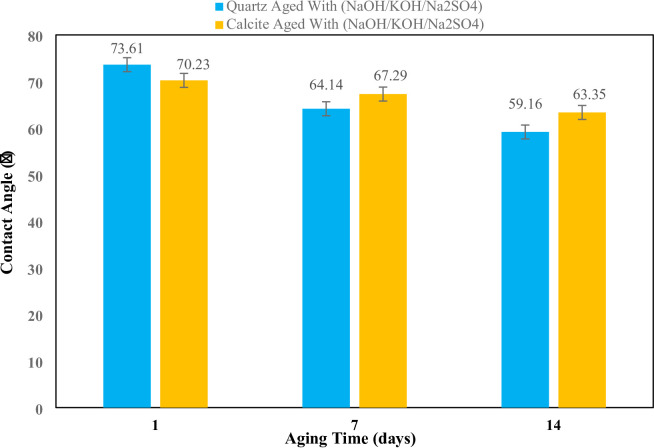
Contact angle response of quartz and calcite to alkaline and Na₂SO₄.

**Fig. 29:**
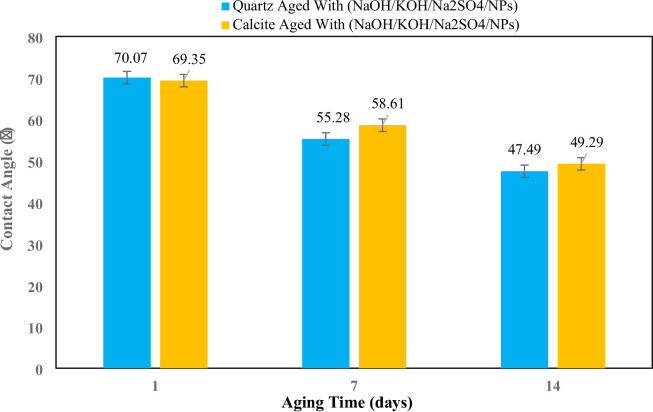
Combination of alkaline and Na_2_SO_4_ and NPs on the contact angle for quartz and calcite.

- **Without nanoparticles**:
- As shown in Fig. 28, after 14 days, the contact angle decreased to 59.16° for quartz and 63.35° for calcite.

- **With nanoparticles**:
- As shown in Fig. 29, the combined system of alkali, salt, and nanoparticles further reduced the contact angle to 47.49° for quartz and 49.29° for calcite after 14 days.

**Fig. 30:**
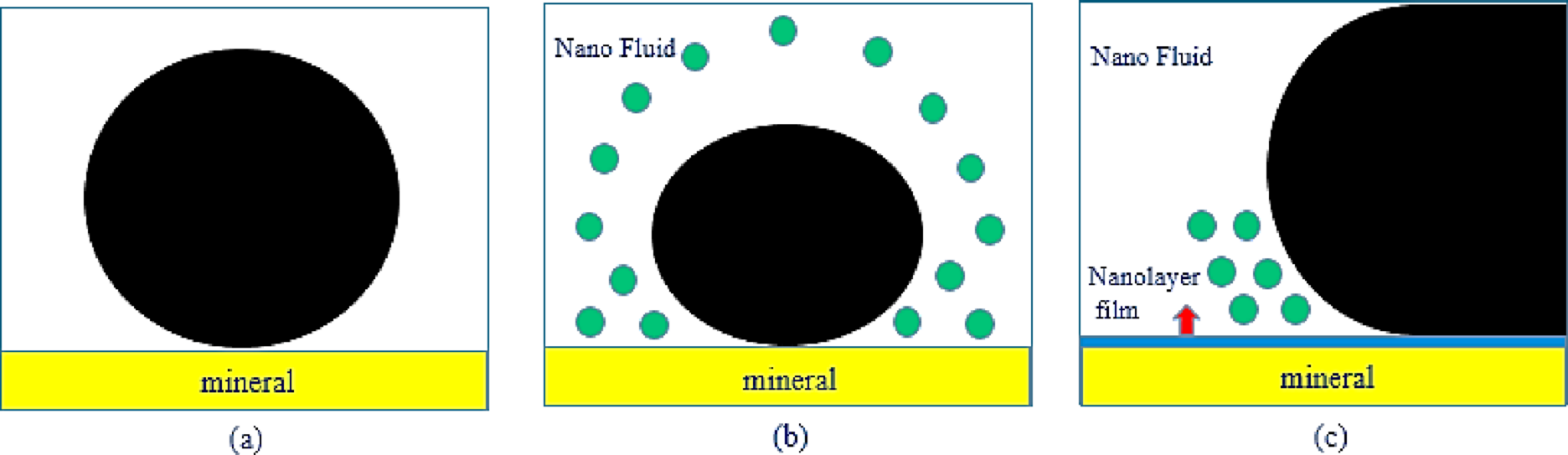
Schematic mechanism of wettability alteration by: (a) oil-wet (b) effect of NPs in wettability rock surface and (c) create nanolayer film with NPs.

Modifications in salt concentration and ionic makeup have an impact on the electrical double layer as well as interfacial ionic interactions, thereby modifying surface charge equilibrium and reducing contact angle under optimal conditions^106^. Nanoparticles contribute through adsorption, structural disjoining pressure generation, and surface free energy modification, facilitating oil film detachment and enhancing water-wetness^97,102,104^. The mechanism of wettability alteration in nanofluids is predicated on structural disjoining pressure. An increase in this disjoining pressure causes the tip of the wedge structure to expand, thereby enhancing nanoparticle dispersion. Ultimately, the nanoparticle-laden aqueous layer displaces the oil adhered to the surface by applying force and reducing the system’s free energy. Figure 30 illustrates the schematic mechanism of rock surface wettability alteration using oil and nanoparticles. The wettability characteristics of the rock surface are altered, moving away from water-wet toward oil-wet conditions. Subsequently, with the addition of nanoparticles, the constructed nanolayer film shifts the wettability closer to water-wet state. The figure also depicts the mechanism governing wettability modification through the application of nanoparticles. Furthermore, it illustrates the self-assembly of a nanolayer, facilitated by these nanoparticles, which effectuates a transition in surface wettability from an oil-wet state to water-wet state^58,110,111^. 

**Fig. 31:**
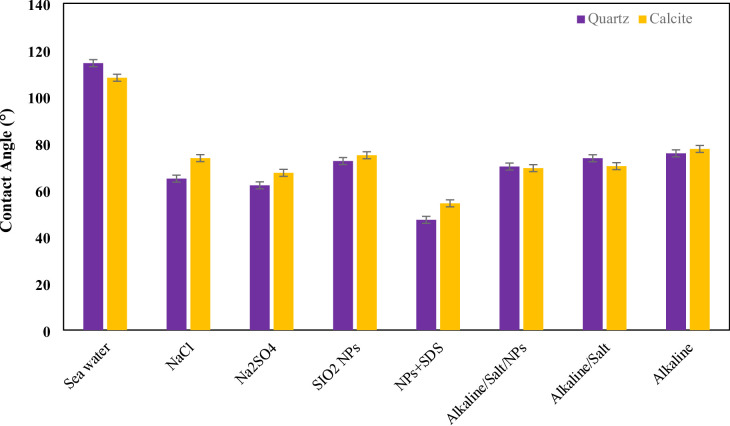
Comparative illustration of contact angle of minerals after 1 day aging.

**Fig. 32:**
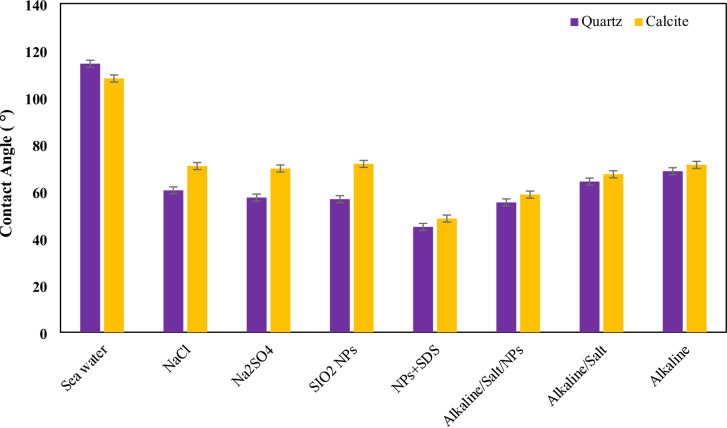
Comparative illustration of contact angle of minerals after 7 days aging.

**Fig. 33:**
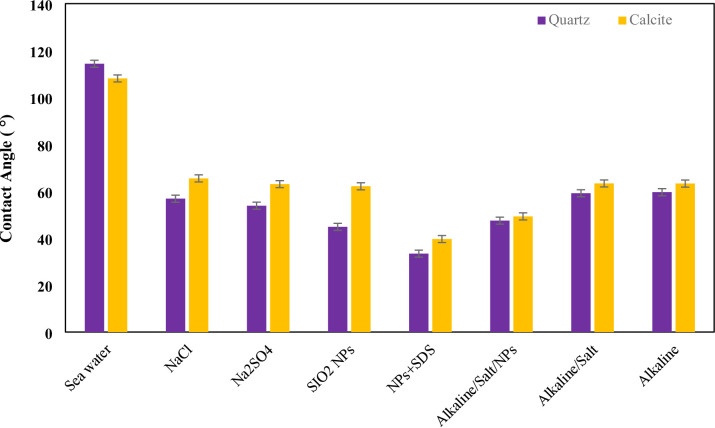
Comparative illustration of contact angle of minerals after 14 days aging.

In the following section, with reference to Figs. 31, 32 and 33, the contact angle results after 1, 7 and 14 days are presented for quartz and calcite surfaces treated with various materials.

**Fig. 34:**
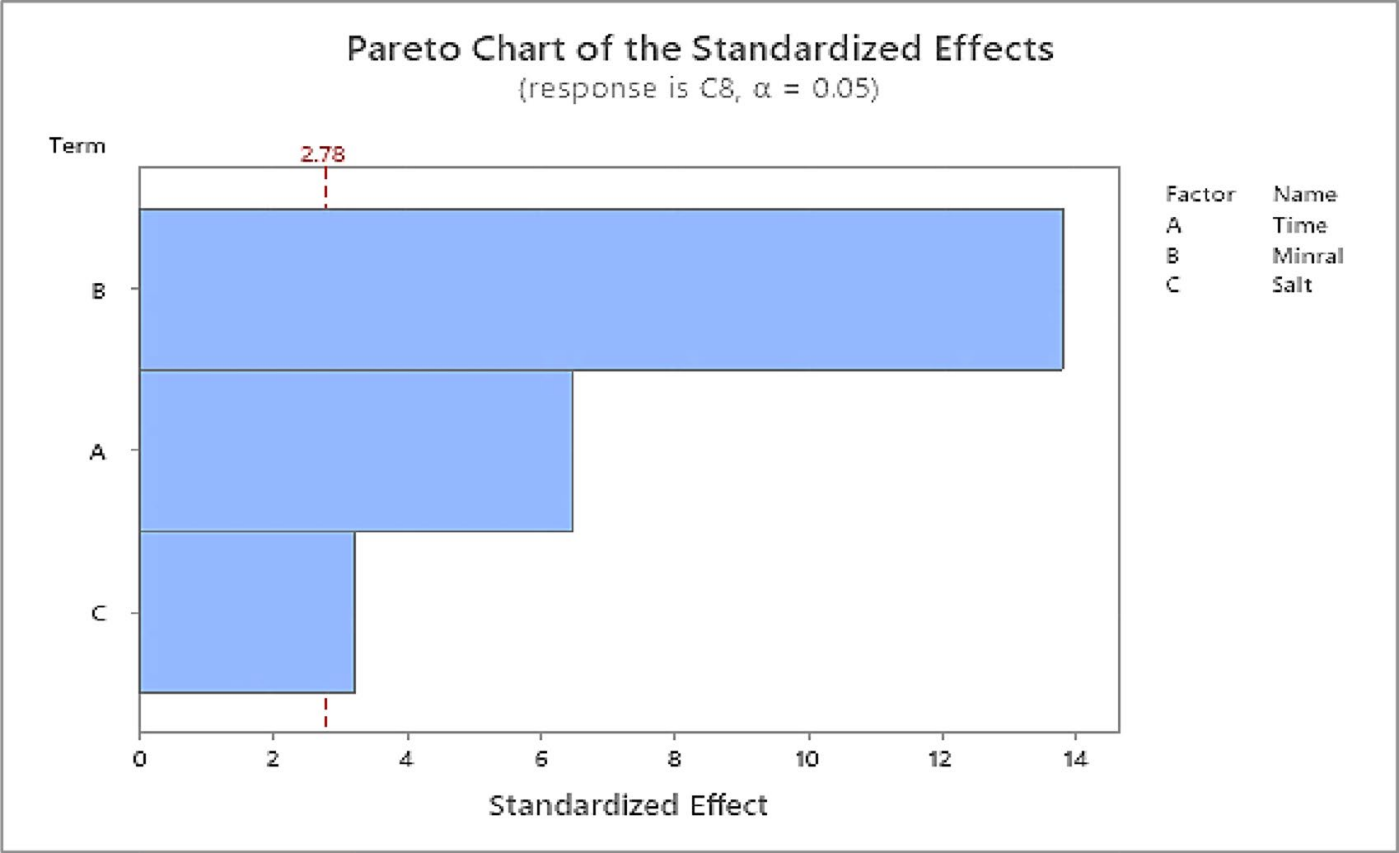
Pareto chart of standardized effects.

The experimental design for the wettability tests and the evaluation of the influencing factors was carried out as illustrated in Fig. 34. The Pareto chart was constructed to evaluate the parameters influencing the contact angle experiments. According to this analysis factor B (mineral) exerts the greatest influence on the response variable. Factor A (time) ranks second, showing a noticeable but smaller effect compared to factor B. Factor C (salt) demonstrates the lowest impact among the examined factors. Since the standardized effects of all three factors exceed the significance threshold (2.78), their influences on the response contact angle are considered statistically significant.

Furthermore, the results of the analysis of variance (ANOVA) are provided in the corresponding Table 5, where each parameter has been comprehensively evaluated and discussed in detail.

**Table 5:** Analysis of variance for the Contact angle test.

source	DF	Adj SS	Adj MS	F-Value	*P*-Value
Model	3	263.148	87.716	80.97	0.000
Linear	3	263.148	87.716	80.97	0.000
Time	1	45.381	45.381	41.89	0.003
Mineral	1	206.396	206.396	190.53	0.000
Salt	1	11.371	11.371	10.50	0.032
Error	4	4.333	1.083		
Total	7	267.481			

**Fig. 35:**
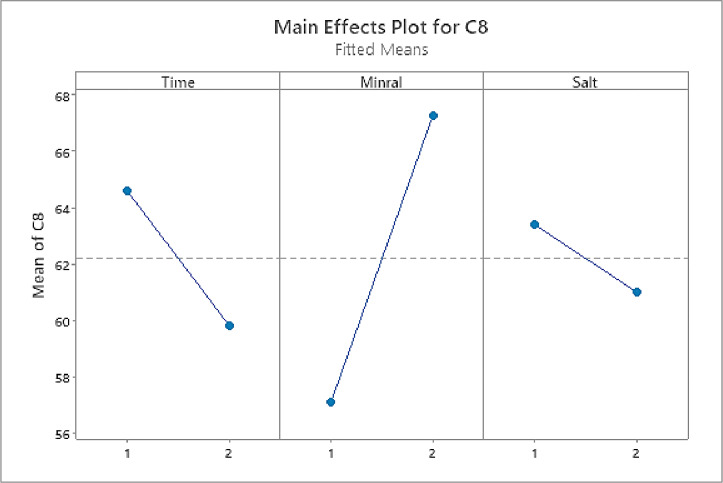
Graph illustrating the effective parameters influencing the contact angle experiments.

The factors affecting contact angle, including contact time, salt type, and mineral type, were investigated at distinct levels. As depicted in Fig. 35, the optimal conditions for influencing the contact angle were identified as: a contact time of 14 days, the use of sodium sulfate (a divalent salt), and the selection of calcite as the mineral.

### Field aspects and challenges of alkali and nanoparticles

Although alkaline flooding offers significant advantages in chemical enhanced oil recovery (EOR), several technical and operational challenges exist. One major limitation is the formation of precipitates due to reactions between alkali and divalent ions such as Ca^2+^ and Mg^2+^ in formation water, which may reduce reservoir porosity and permeability^37,105,106^. Additionally, alkali consumption through reactions with reservoir minerals and crude oil acidic components can negatively impact economic feasibility^27,105,109^.

Process efficiency strongly depends on formation water salinity and hardness. High salinity may reduce IFT reduction performance and limit in-situ surfactant generation. Stable oil-water emulsions may also complicate surface separation processes and increase operational costs. Equipment corrosion under alkaline conditions and uncertainties in scaling laboratory results to heterogeneous field reservoirs represent additional challenges^16,109,116^.

In nanoparticle-assisted EOR, one of the major challenges is establishing a reliable correlation between laboratory-scale results and field performance. Laboratory experiments are typically conducted under ideal and controlled conditions that do not fully represent complex reservoir environments. While studies show that nanoparticles exhibit stability at low temperature and salinity, Their response to challenging downhole conditions, including high temperature, high pressure, and variable salt content, has yet to be fully characterized^35,58,105^.

Furthermore, nanoparticle adsorption mechanisms on different reservoir rock types and the influence of temperature and salinity on this process are not yet fully clarified. Bridging the gap between laboratory findings and field-scale applications remains a critical challenge, requiring comprehensive studies under conditions closely simulating actual reservoir environments. In an alkaline environment, nanoparticles with a high negative charge on their silicon-oxygen bonds form a nanostructured layer at the interface^5,111,117^. This layer reduces the surface free energy. Subsequently, when these nanoparticles are positioned at the interface, they impede the growth of oil droplets, a mechanism akin to pickering emulsion stabilization. By lowering the surface free energy and forming a robust interfacial layer, the IFT is reduced. At the rock surface, the presence of nanoparticles in an alkaline medium induces greater electrostatic repulsion^110,117^. This repulsion aids in the detachment of the thin oil film from the rock. Furthermore, The adsorption of nanoparticles onto the mineral surface leads to the formation of a nanofilm. This layer diminishes the adhesion and intermolecular interactions at the rock-oil interface. As the oil detaches from the rock surface, the contact angle decreases, ultimately enhancing water flow within the porous medium.

### Effect of pH

As a primary controlling parameter, pH regulates chemical interactions in the oil-water-rock system and plays an essential part in enhancing oil recovery. Injection of alkaline agents increases the pH of the injected fluid, creating favorable conditions for the reaction between hydroxide ions and organic acids present in crude oil. Consequently, natural soaps (in-situ generated surfactants) are formed, significantly decreasing the oil-water IFT and enhancing the mobility of trapped oil within the porous media. In this research, the concentration of alkalis was fixed at 10,000, while variations were introduced solely in the type of alkali employed, specifically sodium hydroxide versus potassium hydroxide. Due to this experimental design, the pH values did not undergo significant changes, consistently falling within the 13–14 range. Therefore, pH was treated as a controlled, constant parameter for the duration of the study.

## Conclusion

From the results of this study, it can be pointed out that:

1. While sodium sulfate has a greater effect on reducing the interfacial tension of heavy oil compared to sodium chloride, NaCl has a greater effect on IFT of light oil.
2.  The synergistic combination of alkaline agents NaOH, KOH and sodium sulfate yielded the most significant IFT reduction for all three crude oils compared to the individual use of salt or alkali.
3. The synergistic effect of silica nanoparticles (SiO₂) combined with the optimized solution yielded the best performance for heavy oil C, reducing the IFT to 0.69 mN/m.
4. The combination silica nanoparticles with sodium dodecyl sulfate (SDS) at optimal concentrations have the greatest effect on wettability. After 14 days, the contact angle reduced of 114.4° to 33.4° for quartz and from 108.1° to 39.7° for calcite. Under these conditions, the combination of alkali, sodium sulfate, and silica nanoparticles brought the contact angle down to 47.5° for quartz and 49.3° for calcite.
5.  Crude oil composition was identified as a key factor influencing IFT behavior. Oil C, containing higher amounts of asphaltene and resins compared to oil A and oil B, exhibited a greater IFT reduction.
6.  The produced in-situ surfactant was anionic in nature with a zeta potential of −54.4 mV and demonstrated good stability due to its significant surface charge and aqueous-based environment.
7. The results of this study should be considered as an initial screening of oil types for the combined use of alkaline and nanoparticle in enhanced oil recovery operations.

## Data Availability

The databank utilized during this research is available from the corresponding author on reasonable request.
